# *Rhodococcus* as a Versatile Biocatalyst in Organic Synthesis

**DOI:** 10.3390/ijms20194787

**Published:** 2019-09-26

**Authors:** Hanna Busch, Peter-Leon Hagedoorn, Ulf Hanefeld

**Affiliations:** Department of Biotechnology, Delft University of Technology, Van der Maasweg 9, 2629 HZ Delft, The Netherlands; H.Busch@tudelft.nl (H.B.);

**Keywords:** *Rhodococcus*, biocatalysis, organic synthesis, enzyme, drugs

## Abstract

The application of purified enzymes as well as whole-cell biocatalysts in synthetic organic chemistry is becoming more and more popular, and both academia and industry are keen on finding and developing novel enzymes capable of performing otherwise impossible or challenging reactions. The diverse genus *Rhodococcus* offers a multitude of promising enzymes, which therefore makes it one of the key bacterial hosts in many areas of research. This review focused on the broad utilization potential of the genus *Rhodococcus* in organic chemistry, thereby particularly highlighting the specific enzyme classes exploited and the reactions they catalyze. Additionally, close attention was paid to the substrate scope that each enzyme class covers. Overall, a comprehensive overview of the applicability of the genus *Rhodococcus* is provided, which puts this versatile microorganism in the spotlight of further research.

## 1. Introduction

The genus *Rhodococcus* belongs to the phylum Actinobacteria and its members are aerobic, Gram-positive, non-motile bacteria showing high GC-contents [1]. Numerous members have been isolated from copious sources such as soil, groundwater, marine sediments, internal organs of insects, diseased and healthy animals, or plants-to name a few [2]. While the vast majority is considered harmless, a few species have shown pathogenic properties leading to foal pneumonia (*R. equi*) or leafy gall disease in plants (*R. fascians*). Starting in the 1980s, their application as a (whole-cell) biocatalyst came to the fore and since then, numerous reports of successful bioconversions have been published with an increasing trend [3]. The longstanding synthesis of acrylamide on a multi-ton scale run at several production sites worldwide is hereby considered as the most outstanding example of a rhodococcal whole-cell process [4,5,6].

*Rhodococcus* members are diverse catalysts that degrade a variety of both natural organic and xenobiotic compounds [7]. Amongst others, the *Rhodococcus* species showed biodegradation potential against short- and long-chain alkanes, halogenated and nitro-substituted aromatic, heterocyclic, and polycyclic compounds [8]. Next to physiological attributes such as a high general tolerance to substrates and solvents [8,9], the metabolic diversity of the genus *Rhodococcus* can be explained by the (*i*) presence and mobility of large, linear plasmids; (*ii*) the multiplicity of catabolic genes; (*iii*) the high redundancy of biosynthetic pathways; and (*iv*) sophisticated regulatory networks of their genomes [1,3,10]. This brands the genus *Rhodococcus* as the strong biocatalytic powerhouse that it is seen as today. The usage of whole biosynthetic pathways from *Rhodococcus* strains in the bioremediation of organic pollutants derived from petroleum such as *o*-xylene has been intensively investigated [11,12] and shown to be successful as was, for example, the lignin degradation catalyzed by *R. jostii* RHA1 [13,14,15]. Here, vanillin, a valuable chemical for food flavoring, was mainly produced [13]. The same strain also displayed biodegradation activity against polychlorinated biphenyls (PCBs) [16]. Additionally, *Rhodococcus* strains were used in desulfurization reactions able to degrade sulfur-containing compounds produced in the treatment of fossil fuels like benzothiophene (BT) or dibenzothiophene (DBT) [10,17].

As shortly displayed, the implementation of whole pathways to degrade numerous compounds is a promising tool with strong future potential application [7,18]. Nevertheless, this review will only focus on *Rhodococcus* as a useful tool in organic synthesis, concentrating on defined enzyme reactions catalyzed by purified enzymes or whole-cells primarily leading to enantioenriched products. It should serve as a comprehensive overview of the state-of-the-art biocatalysis that is feasible with *Rhodococcus*, concentrating on the reaction diversity and the respective substrate scope of each enzyme class described.

## 2. Promiscuous Redox-Reactions in *Rhodococcus*

Oxidoreductases (EC 1) catalyze the electron transfer from one molecule (reductant) to another molecule (oxidant), thereby enabling oxidation–reduction reactions often under the requirement of cofactors like the NAD(P)H serving as an electron donor or acceptor. They make one of the biggest groups and as such, an impressive range of redox-reactions is feasible. Additionally, the genus *Rhodococcus* harbors a large amount of oxidoreductases, some of which are already well-established systems even running at an industrial scale, while others are still being thoroughly investigated and developed.

### 2.1. Alcohol Dehydrogenases/Keto Reductases

Alcohol dehydrogenases (ADHs, EC 1.1.x) catalyze the transformation of primary and secondary alcohols to aldehydes and ketones. A common feature of an ADH-catalyzed reaction is the requirement for a primary oxidant within the active site of the enzyme, which oxidizes the alcohol. This process generally occurs via a hydride abstraction. Nicotinamide cofactors (NAD+, NADP+), pyrroquinoline quinones, or flavins are typical prosthetic groups acting as hydride acceptors that need to be regenerated during the reaction for the overall process to be a success [19]. A nicotinoprotein from *R. erythropolis* DSM 1069 showed activity toward aliphatic and aromatic primary and secondary alcohols whereby primary alcohols were the preferred substrates yielding aldehydes [20]. Another application of ADHs in an oxidative sense is the kinetic resolution of secondary alcohols leading to both ketones and enantiopure alcohols. When applying whole-cells of *R. ruber* DSM 44541, only the (*S*)-enantiomer of racemic mixtures of alcohols was oxidized leaving behind the unreacted (*R*)-alcohol. This proof-of-principle was successfully shown for the substrate sulcatol as well as aromatic and aliphatic alcohols [21]. A *R. erythropolis* mediated kinetic resolution of methyl-nonactate yielded the two enantiomers (+)- and (-)-nonactate in excellent enantiomeric purity [22]. Fascinatingly, the stereoselectivity of the main product was influenced by switching the conditions from aerobic to anaerobic. (+)- and (-)-nonactate are the desired building blocks for the macrotetrolide nonactin.

**Scheme 1:**
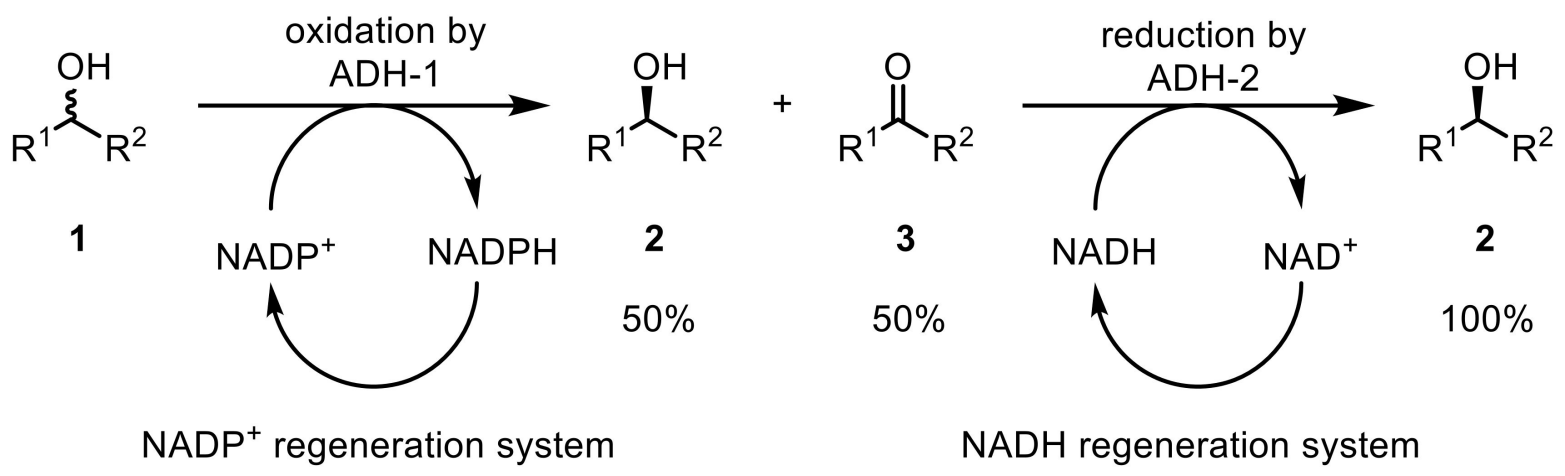
Deracemization of racemic alcohols using a two-enzyme system with stereocomplementary ADHs [23].

To overcome the limitation of only 50% theoretical yields in kinetic resolutions, a two-ADH system was developed resolving racemic alcohols (**1**) [23]. First, one ADH (ADH-1) catalyzes the oxidation of one enantiomer to the corresponding ketone (**3**), leaving one alcohol-enantiomer unreacted (**2**). A second, stereocomplementary ADH (ADH-2) is afterward applied, which reduces the obtained ketone (**3**) to the wanted alcohol-enantiomer (**2**), thereby enabling a theoretical yield of 100% (Scheme 1). Each step is catalyzed by two different ADHs, each requiring another cofactor.

The (*S*)-selective ADH from *R. ruber* DSM 44541 (termed ADH-A) was part of this study leading, in combination with (*R*)-selective ADH from *Lactobacillus kefir* (LK-ADH), to the formation of (*S*)-alcohols in excellent yields and *ee*. A complete stereoinversion of chiral secondary alcohols was also achieved by coupling two stereocomplementary ADHs: while the first ADH oxidized the starting alcohol to the ketone intermediate, a second ADH reduced the ketones to the other alcohol enantiomers, resulting in a complete inversion of the stereochemistry [24].

ADHs from *Rhodococcus* were also employed in an oxidative manner in whole-cell biotransformations for straightforward one-pot multistep reactions [25]. Two representative examples demonstrate the broad applicability of ADHs from *Rhodococcus* within biocascades: the double oxidation of *c*-octane was achieved by a combination of three enzymes, namely a monooxygenase P450 BM3, *Lactobacillus brevis* ADH (*Lb*ADH) and *R. erythropolis* ADH (*Re*ADH) including a cofactor regeneration system, whereby all enzymes were coexpressed in a single host cell [26]. The monooxygenase first oxyfunctionalizes the non-activated *c*-octane to *c*-octanol, which is further oxidized by the two stereocomplementary ADHs. In a similar fashion, four recombinant enzymes (a self-sufficient P450 monooxygenase, two stereocomplementary ADHs (*Lb*ADH and *R*eADH) as well as a ω-transaminase (ATA-117)) were co-expressed in *E. coli*, catalyzing the chiral amination of benzylic compounds such as (substituted)-ethylbenzene in a one-pot two-step process [27].

ADHs that catalyze the reverse reaction are so-called ketoreductases (KREDs). Through carbonyl reduction, the generation of (chiral) alcohols is thereby enabled. As the introduction of chiral centers is seen as more valuable than the loss of enantiomeric centers, this reverse reaction is more often applied in synthetic chemistry. 

The reduction of aldehydes is hereby less often reported due to the fact that this reduction does not invoke a new stereocenter, but leads to primary alcohols. However, a KRED from *Rhodococcus* sp. was employed in the multi-step chemo-enzymatic synthesis of Guerbet alcohols [28]. It was used to catalyze the final step by reducing an aldehyde to form a primary alcohol. The use of enzymes in this example led to an improvement of the overall reaction conditions, reducing the temperature and applied pressure, and increasing the selectivity compared to alternative routes.

While most of the following research is based on the use of KREDs identified from *R. erythropolis* (*Re*ADH) and *R. ruber* (ADH-A), a newly characterized ketoreductase from *R. jostii* TMP1 was applied in the synthesis of chiral alcohols showing a broad substrate acceptance with increased affinity toward aliphatic 2,3-diketones, butan-3-one-2-yl alkanoates, and acetoin as well as the respective derivatives [29]. 

The substrate scope of a recombinantly expressed KRED from *R. erythropolis* was evaluated showing that a multitude of ketones was eligible for KRED-mediated reduction forming the (*S*)-selective alcohols [30]. Next to mono-, di-, and tri-substituted acetophenones, aliphatic ketones have also been implemented in a biphasic reaction medium using an integrated cofactor-recycling system (formate dehydrogenase system). The synthesis of 1-[3,5-*bis*(trifluoromethyl)phenyl]ethanol, another di-substituted acetophenone, which acts as a key intermediate in the synthesis of NK-1 receptor antagonists, was achieved with the same enzyme coupled to another cofactor-recycling system (glucose dehydrogenase system) [31]. In a stirred-tank reactor, space-time yields of 260 g/L per day were achieved. 

**Scheme 2:**
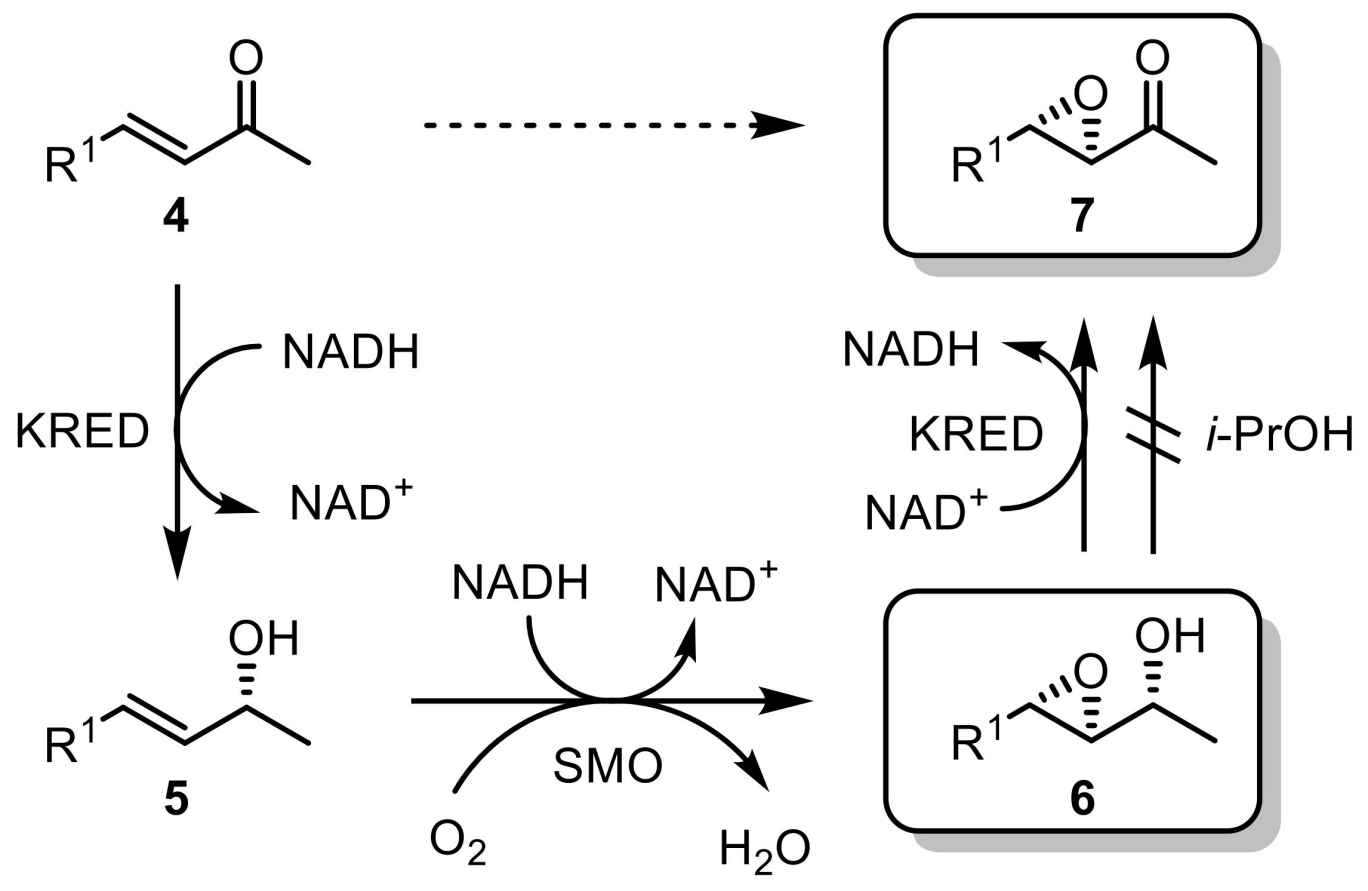
Enzymatic cascade for selective synthesis of epoxy ketones and allylic epoxy alcohols [32].

Interestingly, electron-deficient α,β-unsaturated ketones (**4**) were treated in a tandem reduction–epoxidation–dehydrogenation cascade involving two enzymes, namely an AHD from *R. erythropolis* DSM 43297 and a styrene monooxygenase both co-expressed in *E. coli* [32]. While aiming for the synthesis of chiral epoxy ketones (**7**), it was found that allylic epoxy alcohol intermediates (**6**) also form valuable synthons due to the presence of three contiguous stereocenters. Through adaption of the reaction conditions (the addition of isopropanol, which caused high concentrations of NADH, thereby blocking the dehydrogenation reaction), the major product could easily be switched from an epoxy ketone (**7**) to an allylic epoxy alcohol (**6**), thereby providing two useful synthons with only one reaction set-up (Scheme 2).

**Scheme 3:**
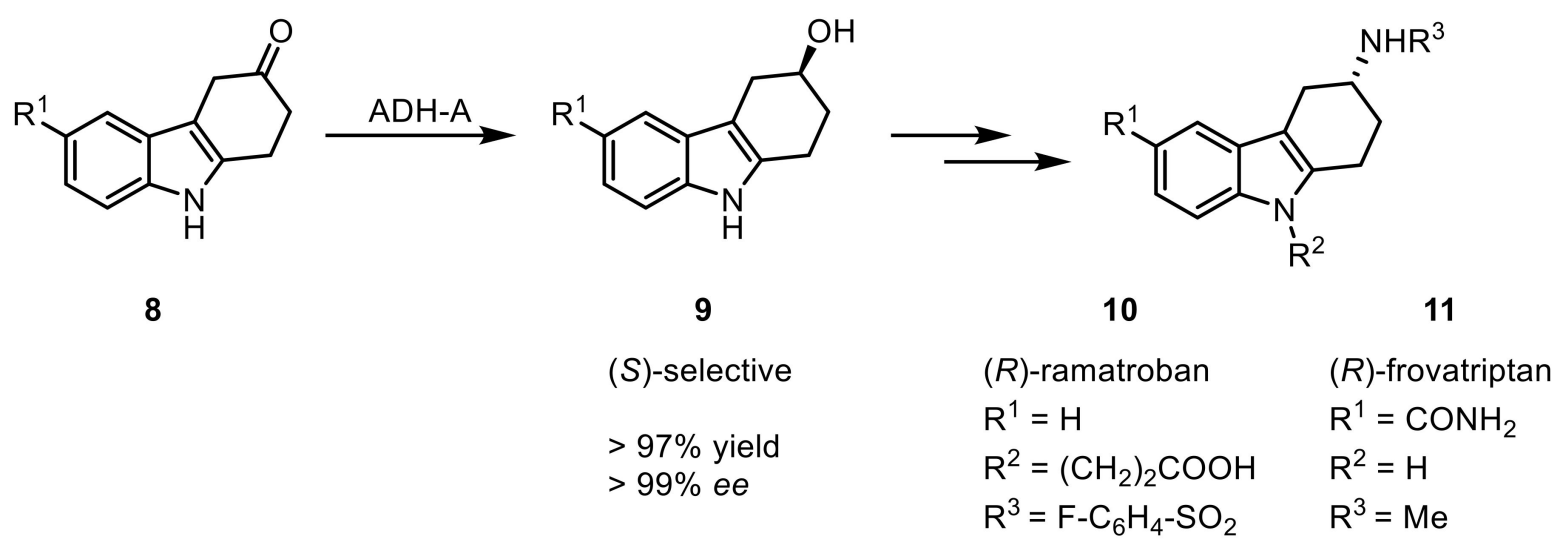
KRED catalyzed ketone reduction in a multistep chemo-enzymatic reaction sequence to pharmacologically active compounds (*R*)-ramatroban and (*R*)-frovatriptan [35,36].

Additionally, trifluoroacetyl-acetophenones lead to chiral fluorinated hydroxyketones when converted by *Re*ADH [33]. The conversion of phenylacyl halides, here especially fluorides, was performed with recombinantly expressed ADH-A yielding fluorohydrins with high yields and enantiomeric purity [34]. ADH-A was also applied in the synthesis of pharmacologically active compounds (*R*)-Ramatroban (**10**), a thromboxane receptor and protaflandin DP2 receptor antagonist, and (*R*)-Frovatriptan (**11**), a 5-hydroxytryptamine (serotonin) receptor antagonist, treating allergic rhinitis and asthma or migraine headaches, respectively [35,36]. In both total syntheses, the KRED was used to install an (*S*)-alcohol (**9**), which was subsequently inverted following a S_N_2-mechanism to an (*R*)-amine. Afterward, the selective functionalization of the amine allowed generation of both desired compounds (Scheme 3).

**Scheme 4:**
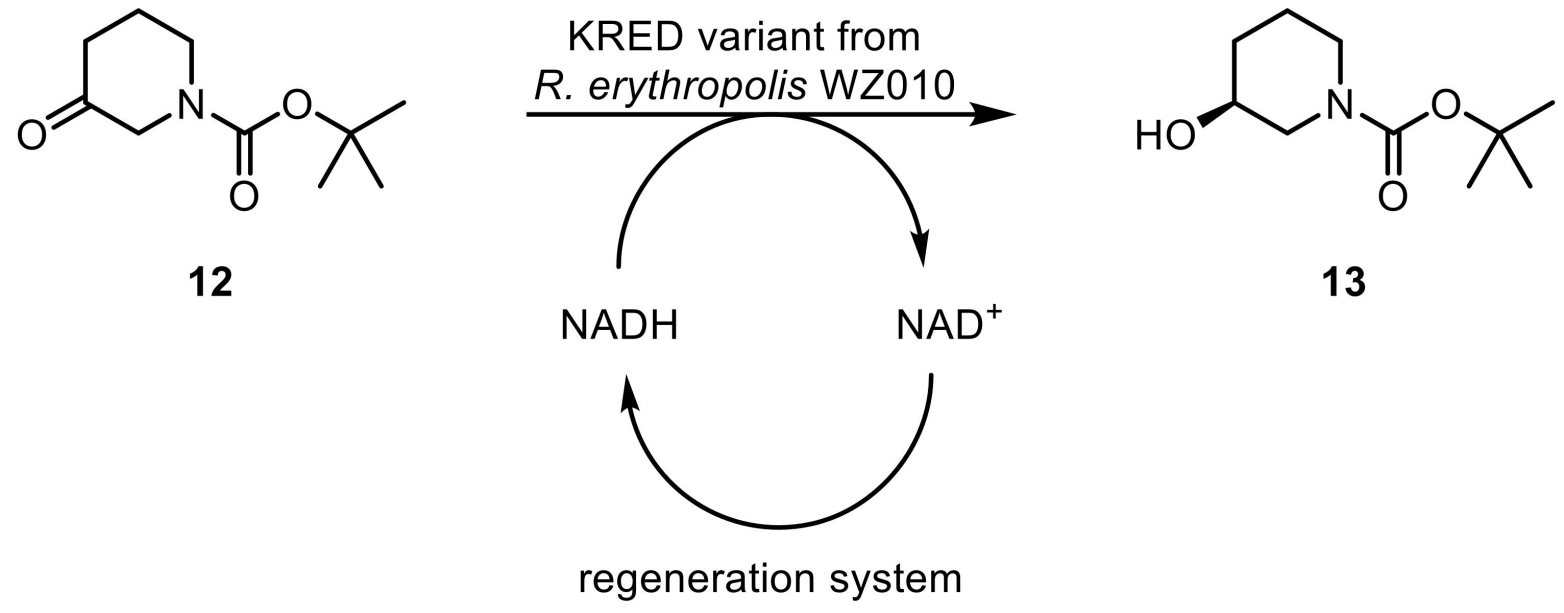
Asymmetric bioreduction of *N*-Boc-3-piperidone (**12**) catalyzed by KRED [39].

The same enzyme (ADH-A) was applied in the production of *syn*-α-alkyl-β-hydroxy amides through means of dynamic kinetic resolution. Various acyclic α-alkyl-β-keto amides were reduced, yielding the (*S*)-selective products’ high yields and excellent *ee* [37]. Enantiopure 3,4-dihydroisocoumarins were easily accessible via a one-pot dynamic reductive kinetic resolution process catalyzed by ADH-A [38] as was the asymmetric synthesis of (*S*)-*N*-Boc-3-hydroxypipridines (**13**) using a variant (Y54F) of a KRED from *R. erythropolis* WZ010 [39]. *N*-Boc-3-piperidone (**12**) was selectively reduced, thereby forming an important intermediate of ibrutinib, an inhibitor of Bruton’s tyrosine kinase. The enzymatic process enhanced the yield significantly when compared to the chemical route (Scheme 4).

The discussed examples show the broad applicability of ADHs/KREDs in organic synthesis by opening up the possibility of introducing new stereocenters through reduction processes or (dynamic) kinetic resolutions.

### 2.2. Oxidases

Another class of enzyme, alcohol oxidases, also catalyze the oxidation of alcohols to aldehydes, ketones, and sometimes carboxylic acids. In comparison to alcohol dehydrogenases that catalyze this reaction via an electron-transfer to an organic cofactor, alcohol oxidases transfer electrons to molecular oxygen forming hydrogen peroxide as a side-product. Using oxygen as a cheap and readily available oxidant makes oxidases more interesting than alcohol dehydrogenases as they require expensive cofactors in either stoichiometric amounts or respective cofactor regeneration systems [40].

Vanillyl alcohol oxidase (VAO) from *Penicillium simplicissimum* is known to catalyze the oxidation of many 4-hydroxybenzylic alcohols. A homologue of this enzyme was found in *R*. sp. RHA1, which was termed eugenol oxidase (EUGO) [41]. The enzyme was shown to catalyze the oxidation of vanillyl alcohol more efficiently than VAO from *P. simplicissimum*, but 4-alkylphenols as well as 4-(methoxymethyl)phenol were only poorly converted.

Cholesterol oxidases (ChoX), on the other hand, catalyze the oxidation of the C3 hydroxyl-group of cholesterol and an isomerization reaction, ultimately yielding cholest-4-en-3-one [40]. In addition to cholesterol, a cholesterol oxidase discovered in *R. erythropolis* also showed activity toward non-steroidal compounds such as smaller cyclic allylic alcohols with good stereo- and enantioselectivity [42]. In an attempt to produce high-quality cholest-4-en-3-one, the use of an aqueous/organic biphasic system was explored which simplified the production process by improving separation and purification [43]. This led to a final product with a purity of 99.78%, which makes this new process design more approachable for industrial applications.

**Scheme 5:**
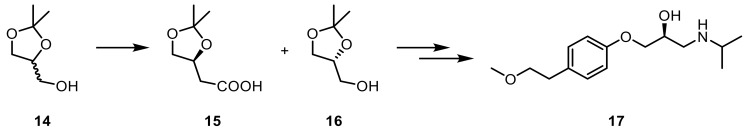
*R. erythropolis* oxidase catalyzed process to produce (*R*)-iso-propylideneglycerol (**16**), which is used as C3-synthon for (*S*)-metoprolol (**17**) [44].

A *Rhodococcus* oxidase catalyzed reaction already running on industrial scale is a kinetic resolution through the oxidation of racemic iso-propylideneglycerol (**14**) yielding both (*R*)-iso-propylideneglyceric acid (**15**) and (*R*)-iso-propylideneglycerol (**16**) [44,45]. The use of this biocatalytic process simplifies the synthesis of desired (*R*)-iso-propylideneglycerol (**16**) compared to chemical processes. Whole-cells of *R. erythropolis* are employed in a fed-batch reactor leading to 50% maximum yield with high *ee* values. The product is a valuable C3 synthon in the synthesis of β-blockers like (*S*)-metoprolol (**17**) (Scheme 5).

### 2.3. Oxygenases in Rhodococcus

Oxygenases utilize molecular oxygen as both a substrate and electron acceptor, while the (above) discussed dehydrogenases catalyze the oxidation via hydrogen transfer reactions. Oxygenases can be classified into two groups: while monooxygenases introduce only one oxygen-atom, dioxygenases catalyze the introduction of two oxygen atoms from molecular oxygen. This type of reaction is particularly useful in synthetic chemistry as a selective activation of chemically inert C–H bonds is otherwise extremely difficult to achieve using classical chemical procedures.

#### 2.3.1. Monooxygenases

##### P450 Monooxygenases

**Scheme 6:**
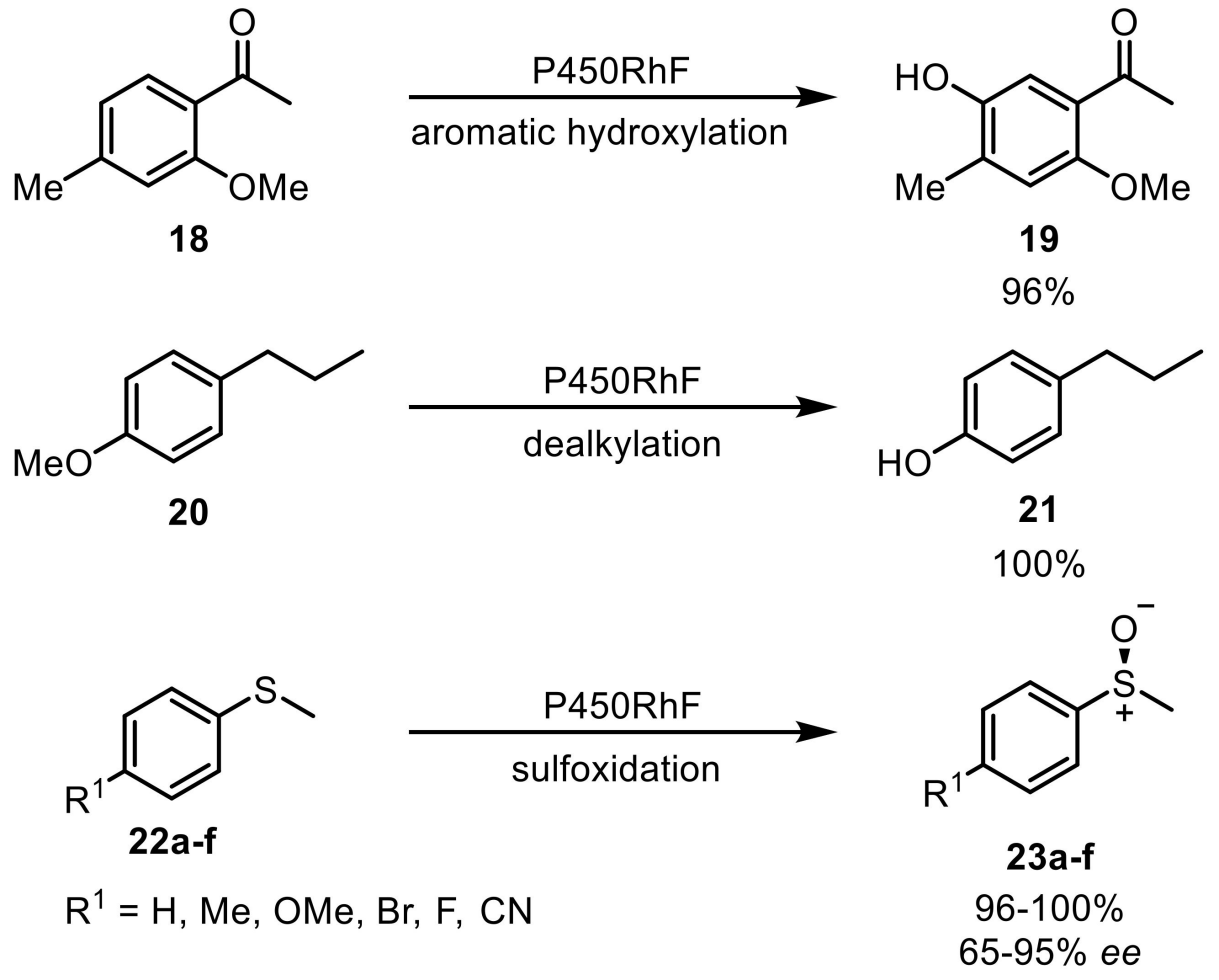
Representative bioconversion catalyzed by P450RhF monooxygenase showing aromatic hydroxylation, dealkylation, and sulfoxidation potential [50].

The so-called cytochrome P450s (P450s, CYPs) monooxygenases contain a heme prosthetic group with an Fe(III)-ion embedded in a porphyrin ring with a cysteine sulfur as an axial ligand to the iron. They activate molecular oxygen for the hydroxylation of organic compounds. In this process, the second oxygen is reduced to water. P450s are NAD(P)H dependent enzymes. Therefore, the redox-reaction required electrons are transferred from the cofactor to the heme through one or two ‘electron transport enzymes’ or ‘redox partners’ [46,47]. This makes P450s multicomponent enzymes. The involved components can either be free or directly linked to each other with the latter being called a self-sufficient enzyme. One of these self-sufficient P450s was discovered in *R*. sp. NCIMB 9784 where the reductase partner (RhFRed) containing the FMN- and NADPH binding motif and a Fe_2_S_2_-ferredoxin-like component is directly linked to the oxidase part [48,49]. The natural substrate of this enzyme has not been discovered yet, but it shows a promiscuous substrate scope mediating dealkylation reactions, aromatic hydroxylation, epoxidation, and asymmetric sulfoxidation (Scheme 6) [50].

The reductase unit of this P450 is, however, more often employed. The heme domain of P450RhF can be swapped with other heme domains, thereby fusing the reductase domain RhFRed to a number of different enzymes [47,51]. One successful example is given by the hydroxylation of testosterone by a chimeric fusion protein consisting of the heme-domain from CYP154 from *Nocardia farcinca* IFM 10152 and the reductase domain RhFRed [51].

##### Baeyer-Villiger Monooxygenases

The Baeyer-Villiger reaction involves the oxidation of a carbonyl compound, ultimately leading to an ester or lactone. So-called Baeyer-Villiger monooxygenases (BVMOs) catalyze this reaction under milder reaction conditions compared to harsh chemical procedures [52]. Flavin-containing enzymes require electrons from a reduced cofactor. The flavin-cofactor reacts with molecular oxygen, thereby forming a reactive peroxyflavin intermediate, which performs the nucleophilic attack on the carbonyl function. Upon a rearrangement, the respective ester or lactone is formed [52].

**Scheme 7:**
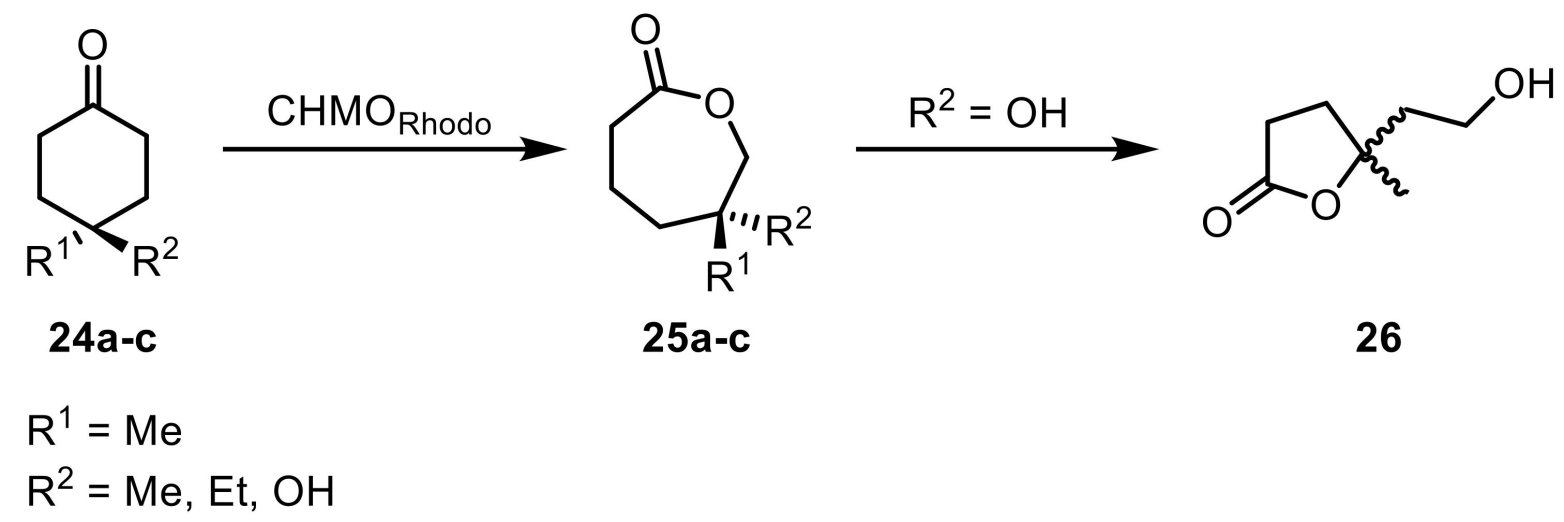
*Rhodococcus* Baeyer-Villiger monooxygenase catalyzed oxidation of 4,4-disubstituted *c*-hexanones (**24**) [55].

Prochiral *c*-butanones with alkyl- or aromatic substituents in the 3-position were converted by the two *c*-hexanone monooxygenases discovered earlier in *Rhodococcus* [53] (CHMO_Rhodo1_ and CHMO_Rhodo2_) to yield (*S*)-selective butyrolactones [54]. Both enzymes displayed especially high activity toward bulky substituent piperonyl and aromatic residues with substituents in the *m*- and *p*-position. The same enzymes were employed in an activity screening toward 4-4-disubstituted *c*-hexanones (**24**) to obtain the respective caprolactones (**25**) [55]. In the special case of 4-hydroxy-4-methyl-*c*-hexanone (**24c**), a caprolactone (**25c**) was generated, which spontaneously formed a five-membered ring (**26**) (Scheme 7).

**Scheme 8:**
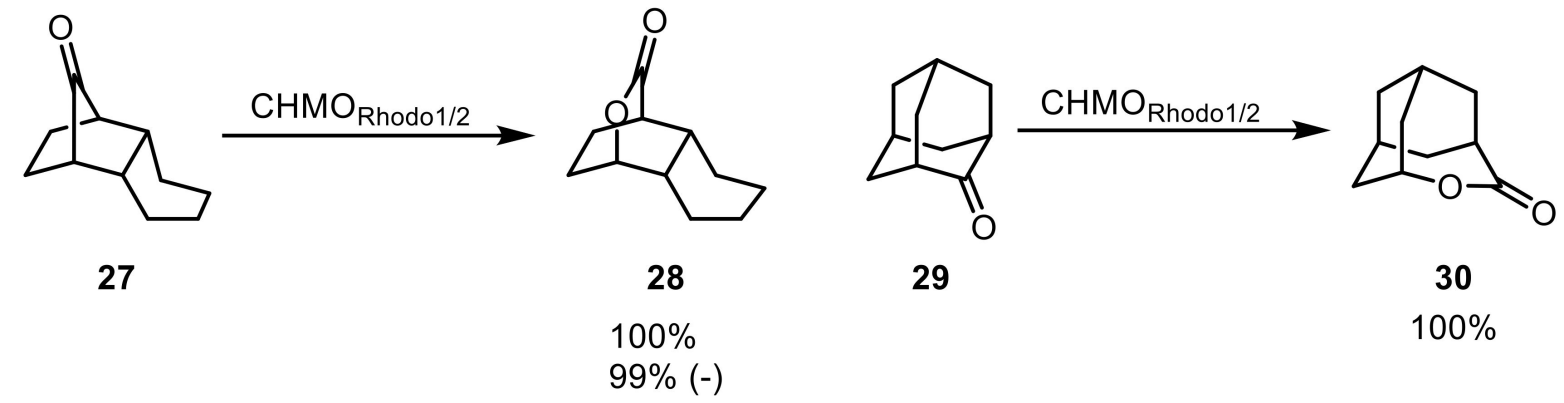
Representative Baeyer-Villiger oxidations of bridged c-ketones (**27**,**29**) catalyzed by CHMOs from *Rhodococcus* [56].

Additionally, the same set of enzymes was used in another screening toward a number of bridged *c*-ketones [56]. Both tested *Rhodococcus* enzymes readily converted almost all tested bridged ketones (**27**,**29**) with high yields and good to excellent stereoselectivities yielding bicyclic lactones (**28**,**30**) (Scheme 8).

The ability to resolve *N*-protected β-amino ketones was investigated using the two *c*-hexanone monooxygenases from *Rhodococcus* amongst other bacterial BVMO [57]. CHMO_Rhodo1+2_ both showed the ability to resolve linear(-branched) aliphatic and aryl-aliphatic 4-amino-2-ketones with a strong preference for middle-chain 4-amino-2-ketones. Longer substrates (C12) were, however, not converted. No activity was detected for 5-amino-3-ketones. Next to the more intensively studied *c*-hexanone monooxygenases from *Rhodococcus* (CHMO_Rhodo1+2_), several novel BVMOs have been identified in various strains. Increasing the number of BVMOs leads to an expansion of the application potential of this useful enzyme class.

**Scheme 9:**
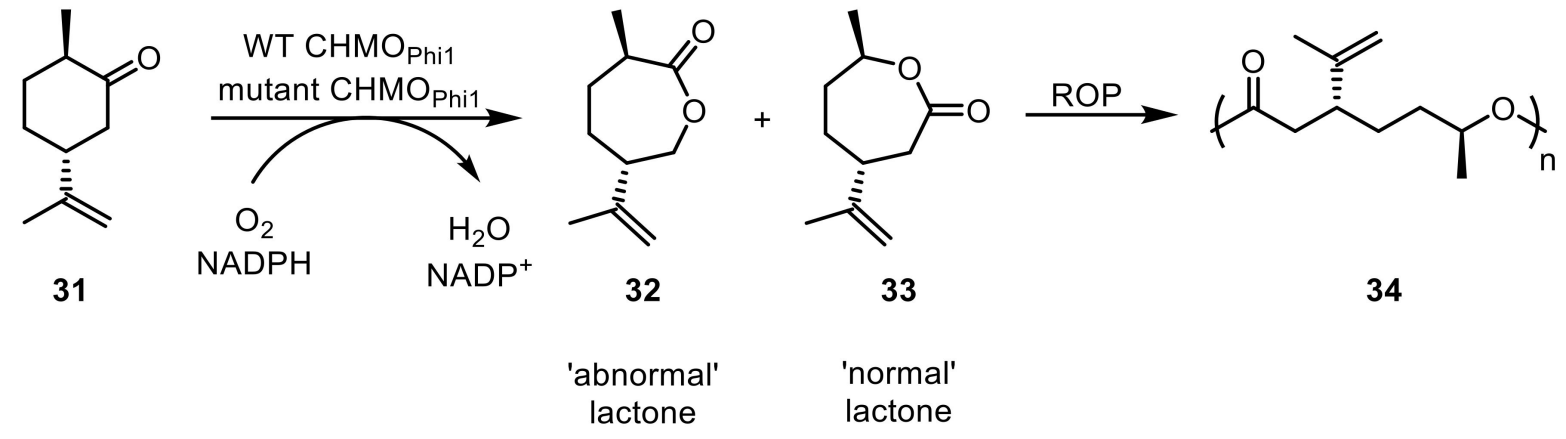
‘Semisynthetic’ production of polydihydrocarvide (**32**/**33**) using a triple mutant of CHMO_Phi1_ [64].

Through a kinetic resolution of 2-(3-penten-1-yl)-*c*-hexanone catalyzed by a *c*-dodecanone monooxygenase from *R. ruber* SC1 (CDMO) [58], the formation of a homologue of the jasmine lactone was achieved with excellent stereoselectivity [59]. Preparation of the jasmine lactone, a desired compound in fragrance industry, was accomplished with a CHMO from *Arthrobacter* in the same study. The species *R. jostii* RHA1 is particularly known for a high abundance of oxidative enzymes [60,61], which gave rise to a comprehensive investigation of the presence of BVMOs in this strain [62]. Following a genome mining approach, a total number of 22 novel BVMOs was identified, expressed, and tested on a diverse set of 39 substrates ranging from linear and cyclic aliphatic ketones to aromatic amines, sulfides, and ketones. Here, in particular, six of the identified BVMOs stood out due to their high substrate promiscuity converting at least 10 and up to 29 of the tested substrates. Furthermore, the same microorganism was shown to also host eight new flavin-containing monooxygenases (FMOs), three of which were successfully applied in Baeyer-Villiger oxidations [63]. Interestingly, the novel enzymes did not favor a specific coenzyme (NADH or NADPH) and their potential as useful biocatalysts was shown by successful conversions of both an aromatic and a bicyclic ketone. A novel CHMO from *Rhodococcus* sp. Phi1 (CHMO_Phi1_) was used in an attempt to chemo-enzymatically produce the lactone monomer dihydrocarvide (**32**/**33**) from monoterpenoid starting materials (**31**) [64]. A subsequent metal-assisted ring opening polymerization (ROP) led to the generation of polydihydrocarvide (**34**), a polymer used as a thermoplastic elastomer. Depending on whether the wild-type enzyme or a triple mutant was employed, the synthesis of so-called abnormal (**32**) and normal lactone (**33**) were favored, respectively (Scheme 9).

In a similar fashion, a *c*-pentadecanone monooxygenase from *Pseudomonas* sp. HI-70 led to the formation of polymenthide in the same study. Finally, a novel BVMO from *R. pyridinivorans* DSM 44555 with extraordinary resistance toward high substrate loading and good stability was shown to convert a number of linear aliphatic ketones [65]. Both 2- and 3-ketones and their respective derivatives were converted. Particularly interesting was the production of 3-acetoxypropionate from methyl levulinate with a space-time-yield of 5.4 g/L per day, thereby more than doubling the highest STY reported thus far.

##### Styrene and Indole Monooxygenases

While the BVMOs are built up by a single-component, the styrene (SMO) and indole monooxygenases (IMO) form their own subgroup within the two-component flavin-dependent monooxygenases [66]. Reduced FAD, which binds in the active site of the monooxygenase, is used for the activation of molecular oxygen and is delivered by a NAD(P)H-dependent flavin reductase. While SMOs consist of a monooxygenase (StyA) and a reductase component (StyB), IMOs can either be built up in the same manner (two-component system with monooxygenase ‘IndA’ and reductases ‘IndB’) or as a self-sufficient fusion protein (ImoA2B) associated with an additional monooxygenase (IndA1).

Styrene monooxygenases catalyze the conversion of styrene and its derivatives and also showed activity against aryl alkyl sulfides. The epoxidation reaction as well as the sulfoxidation reaction solely yielded the respective (*S*)-enantiomers using rhodococcal SMOs with different regeneration systems [67,68,69,70]. Furthermore, the SMO from *R*. sp. ST-10 was used to convert aliphatic alkenes including terminal, internal, unfunctionalized as well as di- and tri-substituted alkenes, thereby generating (*S*)-epoxyalkanes [71]. The same gene was overexpressed in *Kocuria rhizophila* DC2201, a strain with exceptionally high tolerance against organic solvents [72]. This led to an increased conversion yield, thereby making this system a suitable biocatalyst for the environmentally milder production of (*S*)-epoxyalkanes in high purity.

Indole monooxygenases (IndA1 and IndA2B-systems) from *Rhodococcus* species also act on styrene derivatives and catalyze epoxidation and sulfoxidation reactions [67,73,74]. Additionally, SMOs and IMOs were shown to produce indigoid dyes without the formation of byproducts like indirubin [66,75].

#### 2.3.2. Dioxygenases

Aromatic dioxygenases carry out the *cis*-dihydroxylation to arene substrates, thereby generating valuable vicinal *cis*-dihydrodiols. The aromatic ringhydroxylating dioxygenases are non-heme iron-dependent enzymes containing a mononuclear iron-active site and a Rieske type [FeS] cluster for electron transfer. These enzymes also require a ferredoxin and a flavin containing reductase that reacts with NADH. To date, mainly toluene and naphthalene dioxygenases from *Pseudomonas putida* (mutants) have been exploited, but next to those, naphthalene dioxygenases [76] and novel *o*-xylene degrading *Rhodococcus* strains were also discovered [77]. 

In particular, an increased interest on the isolation of the strain *R*. sp. DK17 arose when it was shown to replicate on *o*-xylene and utilize several aromatic compounds (benzene, alkylbenzene, phenol, phthalate), thereby displaying its high degradation potential [11]. Until now, subsequent studies primarily dealt with the deeper understanding of its unique reaction mechanism including the point of initial attack in the arene substrate [77]. In the course of this in-depth investigation, several substrates have already been shown to be modified by either the wild-type or engineered enzyme [78]. Amongst others, the *o*-xylene-3,4-dioxygenase from *R*. sp. DK17 showed, for example, activity against *m*- and *p*-xylene [79] as well as larger substrates such as naphthalene, indan, tetralin and indene, whereby in all cases, the respective *cis*-dihydrodiols were generated [80,81,82].

*R*. sp. I24 was found to be a strain that oxidizes indene via three different enzyme activities: next to a monooxygenase and a dioxygenase both inducible with naphthalene, a toluene inducible dioxygenase is present [83,84]. Great attention has been paid to the bioconversion of indene to *cis*-(1*S*,2*R*)-indandiol, which is a known precursor for (-)-*cis*-(1*R*,2*R*)-1-aminoindan-2-ol, a key chiral synthon for the HIV protease inhibitor Crixivan. Whole-cell experiments with *R*. sp. I24 carried out in a batch- or fed-batch manner struggled with low yields due to the numerous side-reactions catalyzed by the other oxygenases present [83,85]. Therefore, the toluene inducible dioxygenase (TID) was heterologously expressed in *E. coli* and investigated [86]. The desired *cis*-(1*S*,2*R*)-indandiol was produced with an enantiomeric excess of 45.2% over *cis*-(1*R*,1*R*)-indandiol.

Several other *Rhodococcus* species have been investigated for their degradation potential toward the group of BTEXS (benzene, toluene, ethylbenzene, xylene isomers, styrene) aromatics. For example, the conversion of benzoate was catalyzed by a benzoate dioxygenase with a narrow substrate scope from *R. opacus* 1CP [87]. 2,3-dihydroxybiphenyl-1,2-dioxygenases from *Rhodococcus* have been recombinantly expressed, showing activity toward a number of catechols with 2,3-dihydroxybiphenyl being the best accepted substrate [88,89] and several catechol-1,2-dioxygenases were shown to cleave (alkyl-substituted and halogenated) catechols [90,91].

However, to the best of our knowledge, their use as biocatalysts in synthetic chemistry has been limited and not been exploited to its full potential yet.

### 2.4. Miscellaneous Oxidation Potential

As already mentioned, *Rhodococcus* strains show impressive degradation behavior toward a multitude of compounds. Several monooxygenases present in these biodegradation pathways have been identified and implemented in biocatalytic applications. To further showcase the hydroxylation potential of enzymes isolated from *Rhodococcus* as well as whole-cells, several examples are discussed.

With the responsible enzymes staying elusive in some cases, a number of different terpenoids were described to be transformed by whole-cells of *Rhodococcus*. As an example, **d**-limonene was oxidized to (+)-*trans*-carveol by *R. opacus* PWD4 [92] and β-myrcene to geraniol [93].

A 3-ketosteroid-9α-hydroxylase was identified in *R. erythropolis* SQ1, which the 9α-hydroxylation of compounds 4-androstene-3,17-dione and 1,4-androstadiene,3-17-dione [94]. *R. equi* ZMU-LK19 was applied in the asymmetric hydroxylation and diastereoselective oxidation of (+)-2-substituted tetrahydroquinolines generating chiral 2-substituted-1,2,3,4-tetrahydroquinoline-4-ols and chiral 2-substituted-2,3-dihydroquinolin-4(1H)-ones [95].

### 2.5. C=C–Bond Reductases

**Scheme 10:**
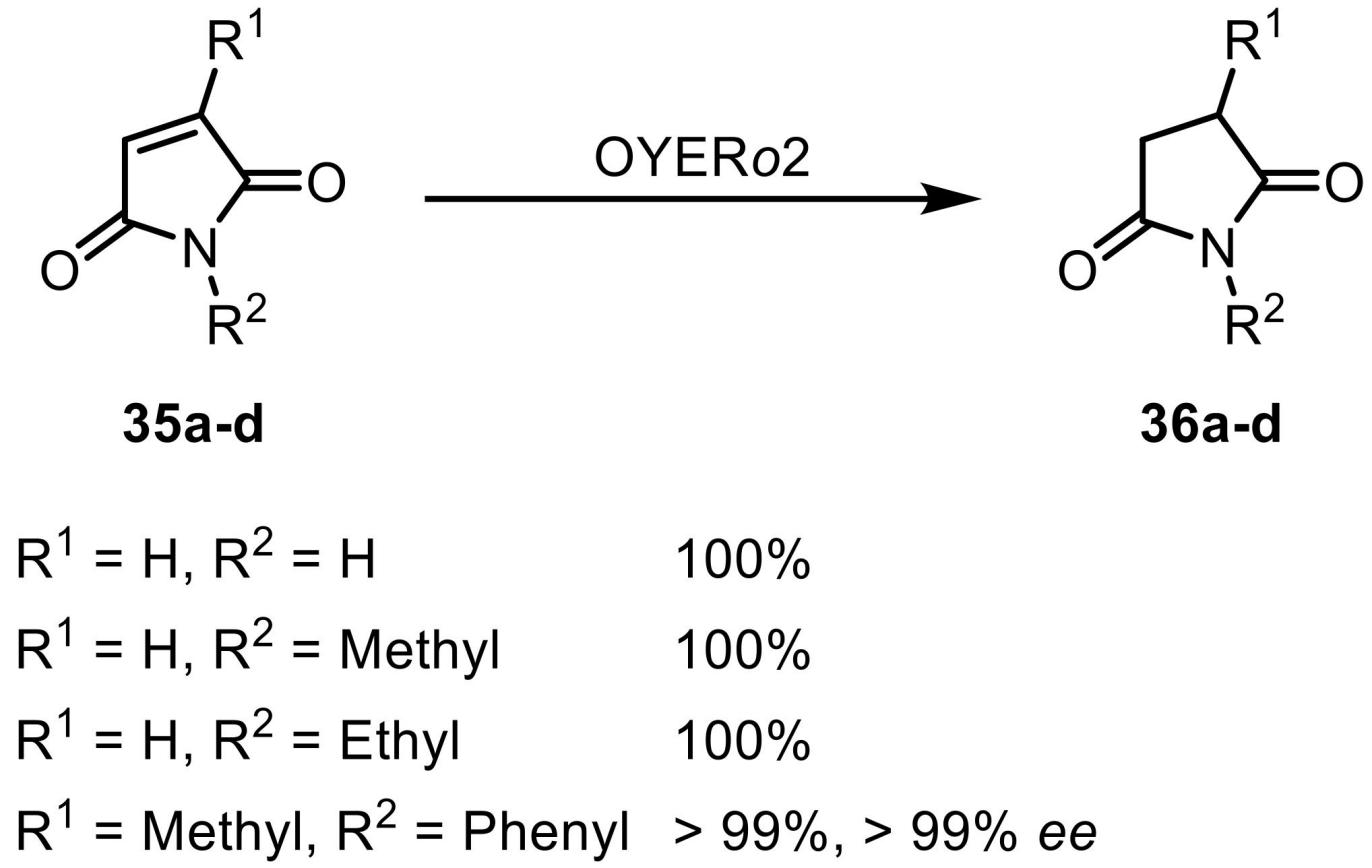
Ene-reductase catalyzed reduction of α,β-unsaturated maleimides (**35**) [99].

The selective reduction of C=C-double bonds, especially in α,β-unsaturated carbonyl compounds, is seen as a valuable reaction in the production of chiral building blocks and is catalyzed by flavin-dependent ene-reductases (EREDs, EC 1.3.1.31, also called Old Yellow Enzymes (OYE) [96]. Like many other reported organisms, *Rhodococcus* also showed the reduction-potential toward a diverse number of substrates. The reactivity toward seven chalcone-derivatives was screened for using *R*. sp. DSM 364 amongst other microorganisms [97]. Whole-cells of *R*. sp. DSM 364 catalyzed the reduction of all seven substrates including all derivatives with both electron withdrawing and electron donating groups, exclusively to the respective dihydrochalcones while leaving the carbonyl moiety unreacted. Several *Rhodococcus* strains (*R. erythropolis* and *R. rhodochrous*) were used in an investigation into their reduction potential toward activated ketones, an aldehyde, an imide, and nitro-compounds [98]. Based on the conversion of ketoisopherone to levodione, stereoselectivity studies have been performed: all so far have reported that ene-reductases found in plants, yeasts, bacteria, and parasites only gave access to the (*R*)-configurated product. However, in this study, whole-cell bioconversions of six out of seven strains led to the formation of the (*S*)-product. The same reaction was carried out with purified ene-reductases, which led to the (*R*)-product. It was therefore proposed that the whole-cells produce a mixture of both (*R*)- and (*S*)-levodione from which only the (*R*)-enantiomer is further converted by the other enzymes present in the whole-cell mixture leaving the (*S*)-levodione as main product unreacted [98]. A (*R*)-selective ‘thermophilic-like’ ene-reductase from *R. opacus* 1CP obtained by genome mining was heterologously expressed in *E. coli* and subsequently characterized [99]. Based on sequence similarity, this enzyme was categorized as a member of the ‘thermophilic-like’ (YqjM-like) OYE group, but it only showed a temperature optimum of 37 °C instead of higher temperatures of ≥70 °C, which are usually described for thermophilic enzymes. It showed the highest activities toward (substituted) maleimides (**35**) leading to the corresponding succinates (**36**) (Scheme 10).

### 2.6. Amino Acid and Amine Dehydrogenase 

Amino acid (AADH, EC 1.4.1.x) and amine dehydrogenases (AmDH) catalyze the reductive amination of α-keto acids and ketones, yielding α-amino acids and amines, respectively, using NAD(P)H as a cofactor. Ammonia is mostly chosen as the nitrogen source. 

*R*. sp. M4 hosts a phenylalanine dehydrogenase (PheDH), which primarily converts phenylpyruvate to **l**-phenylalanine via a reductive amination process [100]. Additionally, the enzyme was also shown to accept other (sterically demanding) α-keto acids such as 4-(methylsulfanyl)-2-oxobutanoic acid, 2-oxo-4-phenylbutanoate or 2-oxo-5-phenylpentanoate, making it interesting for its broad substrate tolerance [100,101]. 

**Scheme 11:**
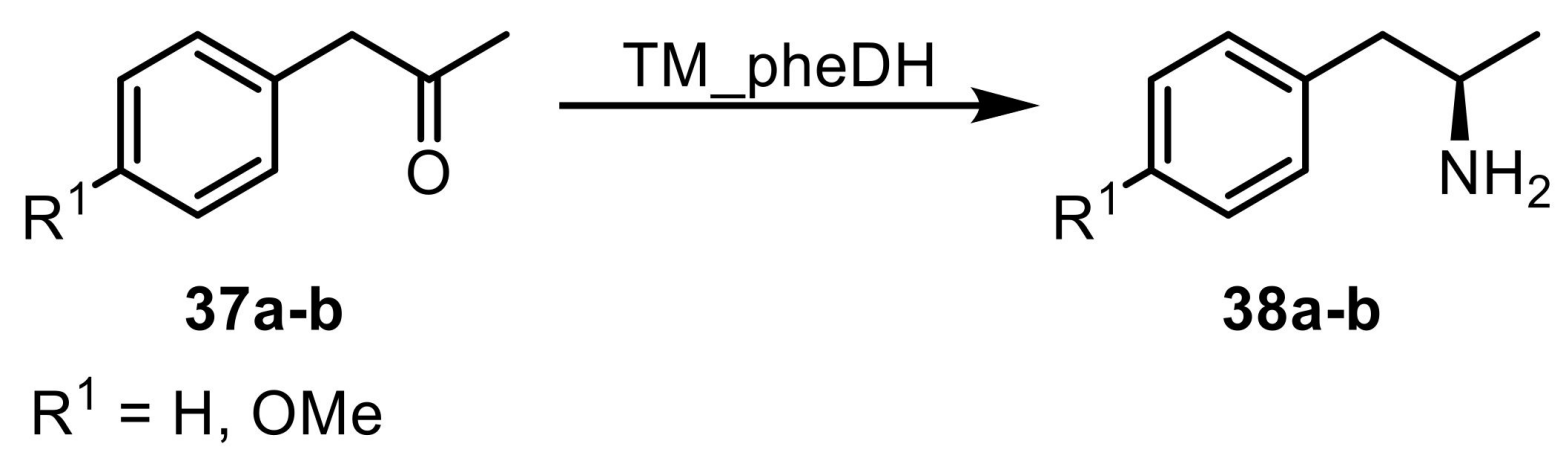
Reductive amination of representative ketones catalyzed by AmDH engineered from *R*. sp. M4 [103,105].

The amine dehydrogenases (AmDH) are a recently developed group of enzymes derived from amino acid dehydrogenases [102]. They act on prochiral ketones, opening up a new synthetic route toward chiral amines. A new (*R*)-selective AmDH (TM_pheDH) was engineered from the *Rhodococcus* phenylalanine dehydrogenase by directed evolution [103]. With this new enzyme, it was possible to reduce phenylacetone (**37a**) and 4-phenyl-2-butanone, leading to (*R*)-amphetamine (**38a**) and (*R*)-1-methyl-3-phenylpropylamine with excellent *ee* values (>98%). The immobilization of this AmDH on magnetic nanoparticles (MNP) increased the productivity and stability compared to the free enzyme [104]. The enzyme was also found to be active toward *o*-methoxyphenylacetone derivatives (**37b**), aliphatic ketones, and so-called ‘bulky-bulky’ ketones such as 1-phenylbutan-2-one or 1-phenylpentan-3-one (Scheme 11) [105].

### 2.7. Desaturase 

A Δ6-desaturase from a *R*. sp. KSM-B-MT66 mutant was applied in a two-phase system to catalyze a *cis*-desaturation of low-cost saturated starting material [106]. Next to unsaturated acyl fatty acids, chloroalkanes and simple alkanes were accepted and the dehydrogenation always took place 9-C-atoms away from the terminal methyl group. This reaction was used to generate intermediates for the preparation of substituted fatty acids used in the dermatological pharmacy and was run with a space-time-yield of 16.8 g/L per day. Recently, novel Δ6-desaturase enzymes were identified in *R*. sp. and their use to produce *cis*-6-hexadecenoic acid were patented for future applications [107,108].

## 3. Enzymes from the Aldoxime-Nitrile Pathway

**Scheme 12:**
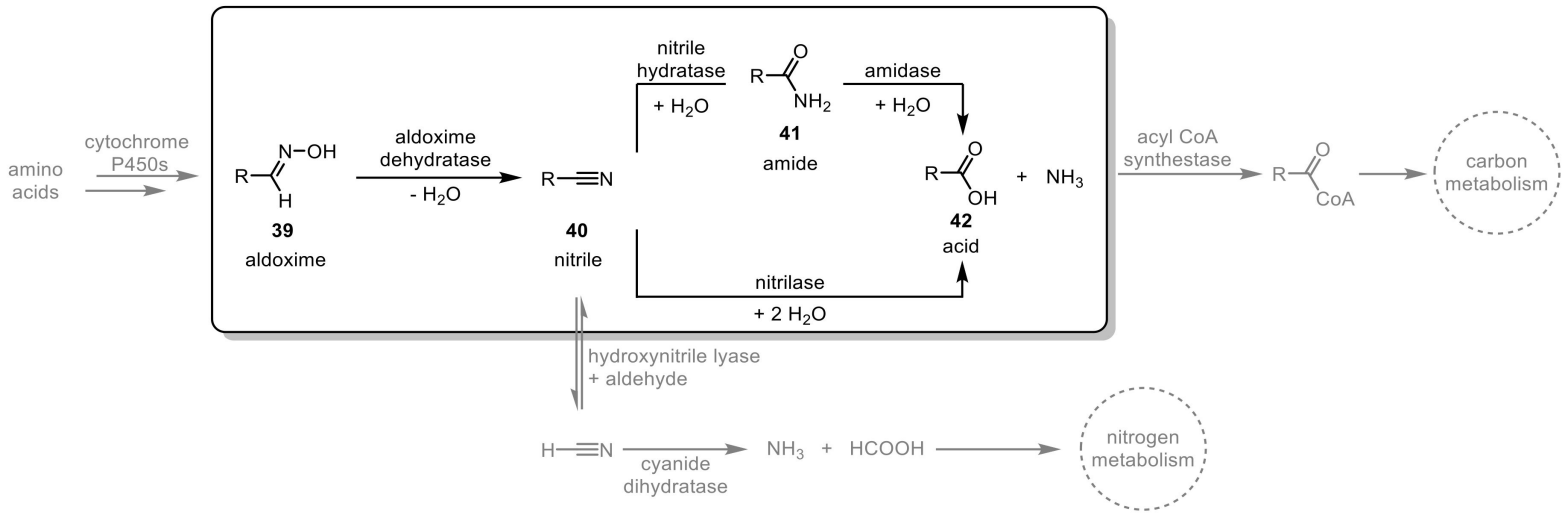
General aldoxime-nitrile pathway in microbes with synthetically used enzymes from *Rhodococcus* highlighted [109].

Enzymes present in the aldoxime-nitrile pathway catalyze both the synthesis and decomposition of nitrogen-containing organic compounds, thereby playing a key role in the carbon and nitrogen metabolism of microbes and plants (Scheme 12) [109,110,111]. Through the oxidation and decarboxylation of amino acids, aldoximes (**39**) are generated [112,113], which are subsequently dehydrated to give nitriles (**40**). These nitriles can either undergo a hydroxynitrile lyase-catalyzed decomposition reaction yielding hydrogen cyanide and aldehydes or they can be converted to carboxylic acids (**42**) via two possible routes: a one-step reaction catalyzed by nitrilases or via an amide intermediate (**41**) catalyzed by a coupled nitrile hydratase and amidase system [109,110]. The resulting acids and ammonia are afterward consumed in the carbon and/or nitrogen metabolism.

Various *Rhodococcus* species use the aldoxime-nitrile pathway and therefore the respective enzymes (aldoxime dehydratase, nitrilase, nitrile hydratase, and amidase) are present in many strains [111,114]. The versatile use of these enzymes in synthetic organic chemistry from a laboratory ‘proof-of-principle’ to multi-ton scale for industrial applications showcases the strength of *Rhodococcus* as a biocatalyst in this area.

### 3.1. Aldoxime Dehydratase 

Nitriles are valuable starting points in the synthesis of both bulk chemicals and chiral pharmaceuticals [96,112]. Their synthesis, however, either requires high temperatures (ammoxidation) or the use of highly toxic hydrogen cyanide as a reagent. Aldoxime dehydratases (Oxd, EC 4.99.1.5) are a recently discovered group of enzymes found in bacteria [115,116] and offer an environmentally friendly, cyanide-free alternative to producing nitriles that start from easily accessible aldoximes, which can be produced by a condensation reaction of aldehydes with hydroxylamine [117]. 

Several studies have examined the substrate scope of aldoxime dehydratases from *Rhodococcus* for non-chiral aldoximes [116,118,119,120]: while arylaliphatic aldoximes were generally better converted by other organisms [121], strain *Rhodococcus* sp. YH3-3 was the only organism that showed activity against substituted aromatic and furan-derived aldoximes. It also displayed higher activities toward a number of substrates with heteroaromatic moieties compared to other Oxds, thereby showing unique properties [112,116]. Linear and branched aliphatic aldoximes with a chain length of C2 to C6 are accepted and converted to their respective nitrile by a number of ROxd [116,118,119]. A comprehensive study compared the activity of five heterologously expressed aldoxime dehydratases of which two originated from *Rhodococcus* strains (*R. globerolus* A-4, OxdRG; *R*. sp. N-771, OxdRE) toward chiral aldoximes including arylaliphatic, heteroaromatic aliphatic, cyclic aliphatic, and acyclic long-chain aliphatic aldoximes with an particular interest in the stereochemical course of the reaction [120]. Interestingly, in some cases, it was shown that depending on the choice of isomeric structure of the substrate (*E*- or *Z*-aldoxime), the enantiopreference in the final nitrile (*R*- or *S*-configuration) could be influenced. 

**Scheme 13:**
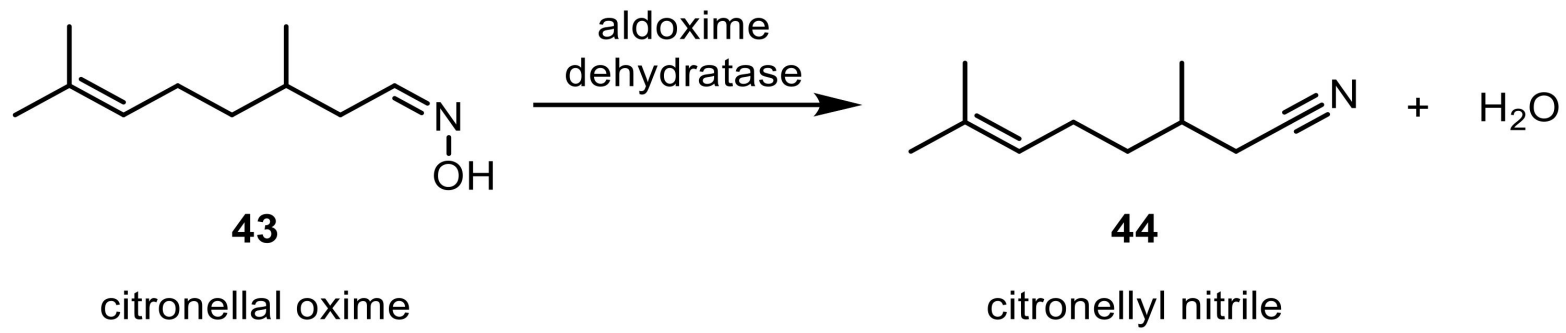
Patented biocatalytic citronellyl nitrile (**44**) production by dehydration of citronellal oxime (**43**) catalyzed by aldoxime dehydratase [122].

A recent example displaying the growing industrial importance of this enzyme class can be seen in a filed patent from BASF, which describes the conversion of a number of terpenes—important odoriferous compounds in the fragrance industry. One example is the biocatalytic production of citronellyl nitrile (**44**), which is known to have a rose-like fragrance (Scheme 13) [122].

### 3.2. Nitrile Hydratase 

While aldoxime dehydratases form valuable nitriles, nitrile hydratases (NHase, EC 4.2.1.84), which are mononuclear iron- or cobalt-dependent enzymes, belong to the group of nitrile-degrading enzymes yielding amides through hydration reactions. This reaction is often followed by an amidase-catalyzed hydrolytic step when applying *Rhodococcus* whole-cells, which converts the formed amides to their respective carboxylic acids. This section will summarize both the use of NHase as a single catalyst and systems using both enzymes as a coupled two-step system. 

#### 3.2.1. NHase as a Single Biocatalyst

The production of amides from nitriles has become crucial to industry and therefore the commercial interest in nitrile-degrading enzymes has gained immense attention, as many excellent reviews display [109,114,123,124]. *Rhodococcus* strains are industrially used to prepare amides essential for humankind such as acrylamide (*R. rhodochrous* J1, >400,000 t/a, Nitto Chemical Industry [5,6]) or nicotinamide (*R. rhodochrous* J1, >11,500 t/a, Lonza AG [45,125]). In this case, the nitrile-hydratase of industrially important strain *R. rhodochrous* J1 was shown to be cobalt-dependent [126]. The main products of acrylamide and its polymers are used as coagulators in the leather and textile industry while nicotinamide is one of the two forms of vitamin B3 used in the cosmetics industry and in animal feed supplementation [109,127].

**Scheme 14:**
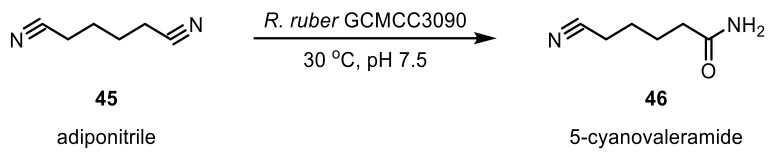
Nitrile hydratase catalyzed bioconversion of adiponitrile (**45**) yielding 5-cyanovaleramide (**46**) using whole-cells of *R. ruber* GCMCC3090 [139].

NHases are versatile enzymes that accept a broad range of different nitriles. The hydrolysis of aromatic and arylalkyl nitriles was intensively studied and proven successful for pyridyl-, pyrazinyl-, (substituted) benzyl-, furyl-, and thionyl-moieties [128,129,130,131,132,133] as well as trans-2,3-epoxy-3-aryl-propannitriles [134] or *rac*-mandelonitrile [135]. *R. boritolerans* FW815 was shown to have a strong 2,2-dimethyl-c-propanecarbonitrile (DMCPCN) hydratase activity in the absence of amidase activity, leading to an enrichment of 2,2-dimethyl-c-propanecarboxamide (DMCPCA)—an important precursor for the drug cilastatin, which is an inhibitor of a renal peptidase that is involved in the metabolism of other drugs, thereby making these other, combined drugs more effective [136]. Dinitriles are also accepted substrates: whole-cells of *Rhodococcus* sp. were shown to convert fluorinated aromatic dinitriles [137] and *R. rhodochrous* IFO 15564 was active toward alicyclic mono- and dinitriles, affording the products in low to moderate yields [138]. Resting cells of *R. ruber* CGMCC3090 converted the aliphatic adiponitrile to selectively give 5-cyanovaleramide (5-CVAM), which is used in the synthesis of caprolactam, a common precursor for Nylon 6 (Scheme 14) [139].

**Scheme 15:**
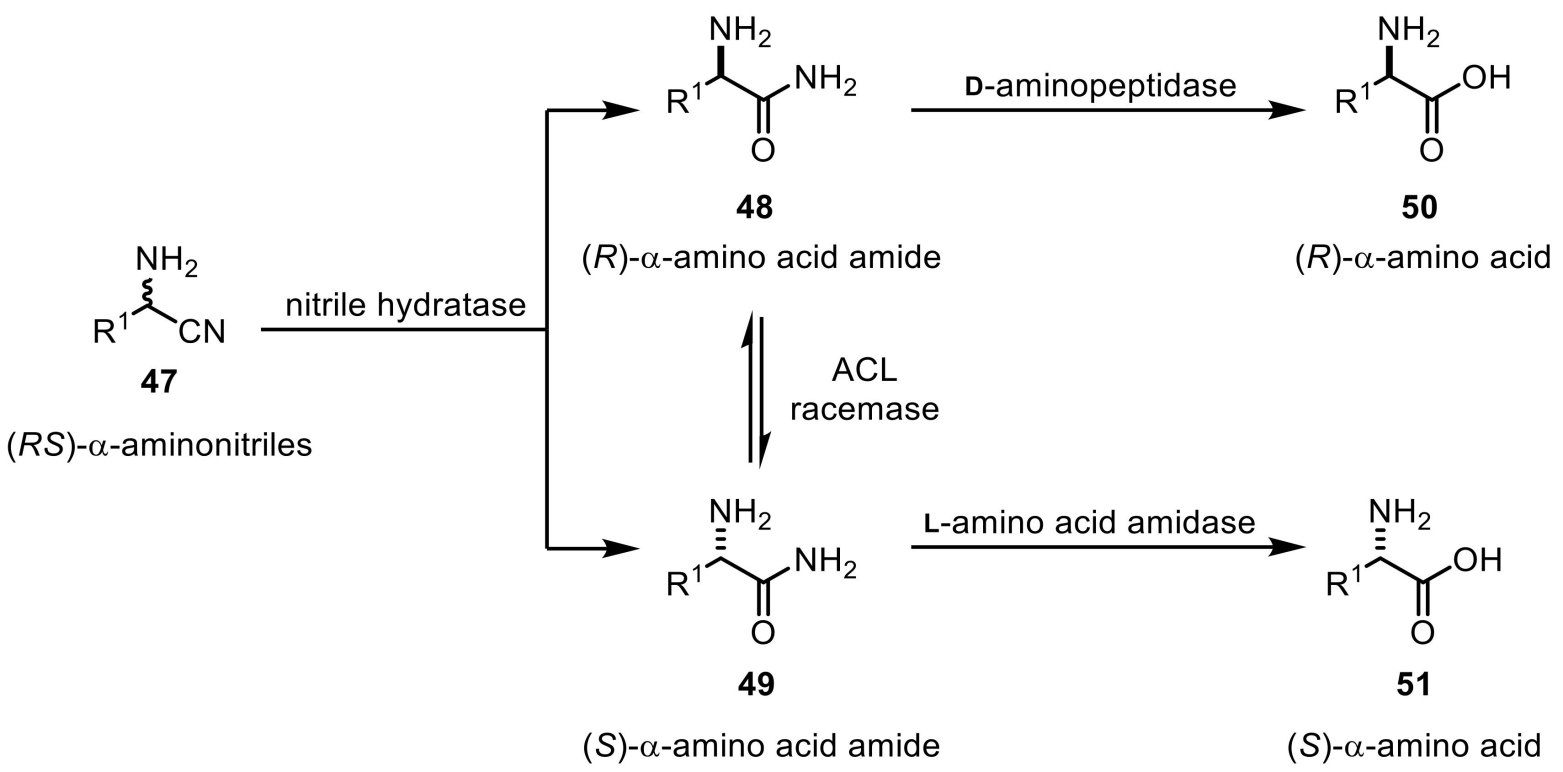
Dynamic kinetic resolution of *rac*-aminonitriles to produce chiral α-amino acids (**50**,**51**). The first step is catalyzed by a nitrile hydratase from *R. opacus* 71D [140].

Prochiral substrates such as α- or β-substituted nitriles have also been used in bioconversions: starting from α-racemic aminonitriles (**47**), respective (*R*)-(**50**) and (*S*)-selective α-amino acids (**51**) were produced each using a three-enzyme cascade reaction [140]. In both reaction pathways, a NHase from *R. opacus* 71D was first applied to give a racemic mixture of α-amino acid amides (**48**/**49**). A dynamic kinetic resolution catalyzed by ACL racemase and subsequently either **d**-aminopeptidase ((*R*)-amino acid (**50**)), or **l**-amino acid amidase ((*S*)-amino acid (**51**)) yielded the final products (Scheme 15).

**Scheme 16:**
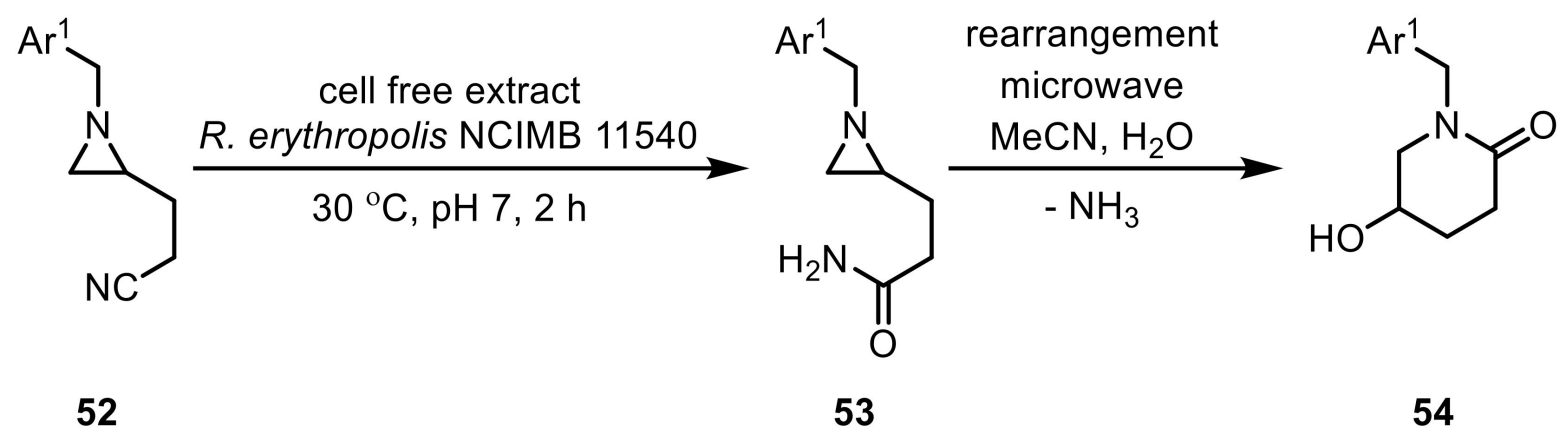
Chemo-enzymatic cascade reaction to synthesize piperidin-2-ones (**54**). The enzymatically catalyzed step was performed by the soluble nitrile hydratase from *R. erythropolis* NCIMB 11540 [142].

On the other hand, a partially purified NHase was successfully applied in the conversion of β-substituted nitriles such as 3-oxonitriles, 3-hydroxynitriles, and 3-(acyloxy)nitrile, yielding the corresponding amides in moderate to good yields [141]. Whole-cells of *R. erythropolis* NCIMB 11540 on the other hand were used in the synthesis of piperidin-2-ones (**54**) starting with a NHase-catalyzed hydration reaction of 1-(arylmethyl)-2-(2-cyanoethyl)aziridines (**52**) (Scheme 16) [142].

#### 3.2.2. Two-Enzyme Systems

**Scheme 17:**
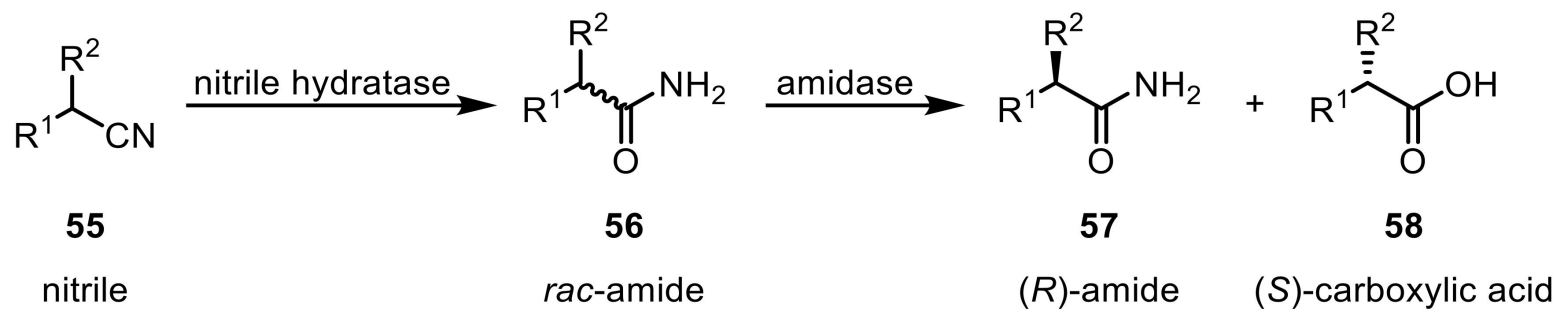
General scheme of the NHase/amidase catalyzed reaction.

Most of the *Rhodococcus* strains used in these bioconversions express both NHase and amidase, which can lead to the direct follow-up reaction catalyzed by amidases yielding valuable carboxylic acids. Nitrile hydratases generally show low stereoselectivities. The amidase hydrolysis reaction, however, is usually very stereospecific, leading to the (*S*) carboxylic acid (**58**) and leaving the unreacted (*R*) amide (**57**) behind (Scheme 17) [143,144]. Many examples have successfully used the presence of both enzymes to achieve the production of both selective amides and carboxylic acids by kinetic resolution.

When starting with prochiral α-substituted nitriles, the synthesis of optically active (*R*)-amides and (*S*)-carboxylic acids was achieved. Several 2-aryl-alkylnitriles were accepted substrates [145,146] as was *rac*-naproxen nitrile, which directly formed the desired and biologically active drug (*S*)-naproxen [147]. Additionally, enantioenriched 3-aryl-3-hydroxy-2-methylene carboxylic acids and amides were prepared from the respective nitriles using whole-cells of *R. rhodochrous* AJ270 [148].

Multiple examples of dinitriles were also applied: incubation of *R. erythropolis* AJ270 cells with *meso*-*c*-pentane-1,3-dinitriles yielded optically active amide-products in high yields [149]. Via an amidase-catalyzed kinetic resolution step and following treatment with CH_2_N_2_, monocyano amides were resolved into (-)-amide and (-)-ester. Additionally, it was shown that the amidase also catalyzes the desymmetrization of *meso*-*c*-pentane-1,3-dicarboxamides, affording enantiopure pentanecarboxylic acids. 

Malononitriles are α, α-substituted dinitriles that form malonic diamides and malonic acid monoamides upon incubation with whole-cells of *R. rhodochrous* IFO 15564 [150]. Next to that, cyanohydrins (α-hydroxy nitriles) were also successfully hydrolyzed, leading to enantiopure α-hydroxy carboxylic acids like the pharmaceutical intermediate (*R*)-chloromandelic acid on gram-scale [151] as was a large variety of aminonitriles ranging from α-aryl-, α-alkyl-substituted glycine nitriles [152,153,154] to aziridine-2-carbonitriles [155], azetidine-2-carbonitriles, and 4-oxoazetidine-2-carbonitriles [156,157]. 

Aside from α-substituted nitriles, β-substituted nitriles are also suitable substrates for the NHase/amidase system, yielding interesting building blocks with more remote stereogenic centers. Next to several studies focusing on 3-substituted glutaronitrile derivatives [158,159,160], 3-hydroxy-arylpropanenitriles [161], 3-hydroxy-4-aryloxybutanenitriles [161,162], and 3-(benzoyloxy)pentanenitrile [163] were also converted using *Rhodococcus* cells. A number of *Rhodococcus* strains was also shown to be active on 3-benzyl-oxy- and 3-benzoyloxypentanedinitriles [88,159,160,161,164], which opens up a new synthetic route to the drug Atorvastatin (trade name Lipitor by Pfizer), an HMG-CoA reductase inhibitor for lowering blood cholesterol [165]. 

The above-mentioned examples all successfully exploit the presence of both enzymes to obtain amides and carboxylic acids. However, the amidase also causes unwanted side-product formation in other reactions when the intermediate amide is the desired final product. To avoid the amidase-catalyzed follow-up reaction in the preparation of acrylamide, an amidase-negative mutant was designed using a genetic knock-out technique. This approach increased the acrylamide yield by 25% compared to the wild-type strain [166].

### 3.3. Amidase 

Amidases (EC 3.5.1.4) belong to the group of amidohydrolases and have been widely researched due to the biological relevance of amides in nature. They generally show a broad substrate scope and good enantioselectivity [167]. The examples discussed in the above section already display the versatility of *Rhodococcus* amidases and their synthetic potential in combination with nitrile hydratases. However, this section highlights specific examples achieved with single-amidase systems. 

An amidase from *R*. sp. MTB5 showed good activity toward a large number of aliphatic, aromatic, and heterocyclic amides [168]. Diamides and specific amino acids were, however, only poorly accepted. α-alkyl phenylacetamides have been kinetically resolved using an amidase present in *R*. sp. AJ270 [169]. (*S*)-naproxen can be produced starting from both racemic naproxen nitrile using the two-enzyme system [147] as well as from a racemic naproxen amide using cells of *R. erythropolis* MP50 [170]. In a similar manner, *R. erythropolis* AJ270 catalyzed the enantioselective hydrolysis of racemic ibuprofen amide leading to the biologically active compound [171]. 

**Scheme 18:**
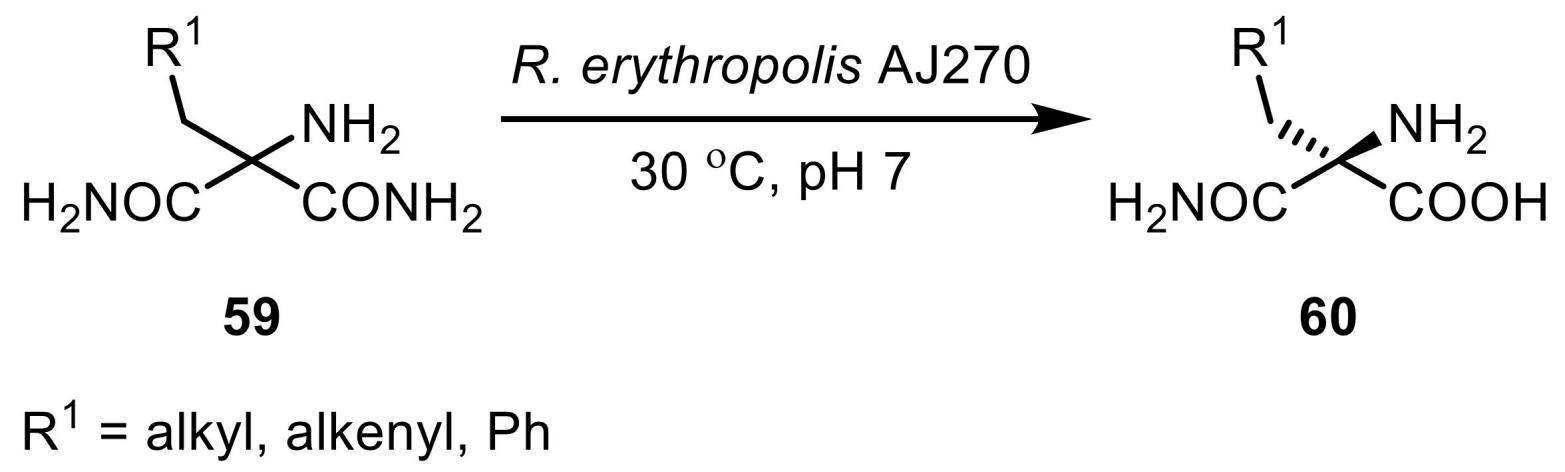
Synthesis of enantioenriched functionalized α-tetrasubstituted α-amino acids (**60**) through amidase-catalyzed hydrolysis [174].

In the production of (*R*)-malonamic acids, amidases from *Rhodococcus* have proven to be successful tools. Whole-cells of *R*. sp. CGMCC 0497 converted aromatic α,α-disubstituted malonamides with substituents in *ortho*-, *meta*-, and *para*-position with high yields and excellent enantioselectivities as well as dialkyl-substituents with slightly lower *ee*-values [172]. The amidase present in whole-cells of *R. erythropolis* AJ270 transformed a number of prochiral malonamides yielding a range of carbamoylacetic acid products [173]. α-substituted α-amino-malonamides (**59**) were transformed to highly functionalized tetrasubstituted α-amino acids (**60**) with high yields with excellent *ee*-values by the same organism (Scheme 18) [174].

**Scheme 19:**
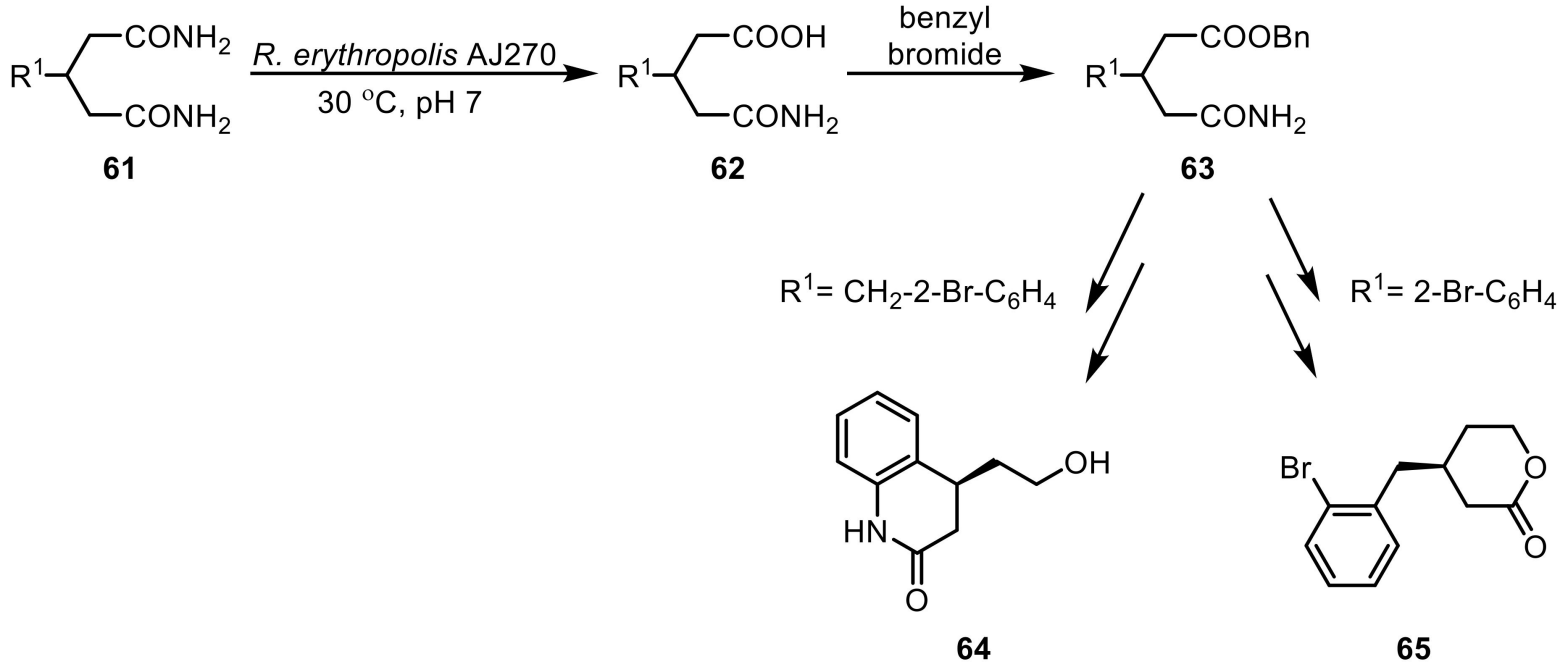
Chemo-enzymatic syntheses of dihydroquinolinone (**64**) and δ-lactone (**65**) [177].

Interestingly, this organism was also used to catalyze the desymmetrization of both five-membered *meso*-*N*-heterocyclic- and *meso*-*c*-pentane dicarboxamides to afford functionalized enantiopure derivatives of pyrrolidine, dihydropyrrole, and piperidine as well as *c*-pentanecarboxylic acids [175,176]. The same amidase was reported to also hydrolyze a palette of prochiral 3-aryl- and 3-arylmethyl glutaramides (**61**), leading to highly enantioselective glutaric acid monoamide derivatives (**62**) [177]. This amidase-catalyzed hydrolysis paved the way for a straightforward chemo-enzymatic synthesis of dihydroquinolinone (**64**) and a δ-lactone (**65**), thereby showcasing the many synthetic applications of this biocatalyst (Scheme 19).

**Scheme 20:**
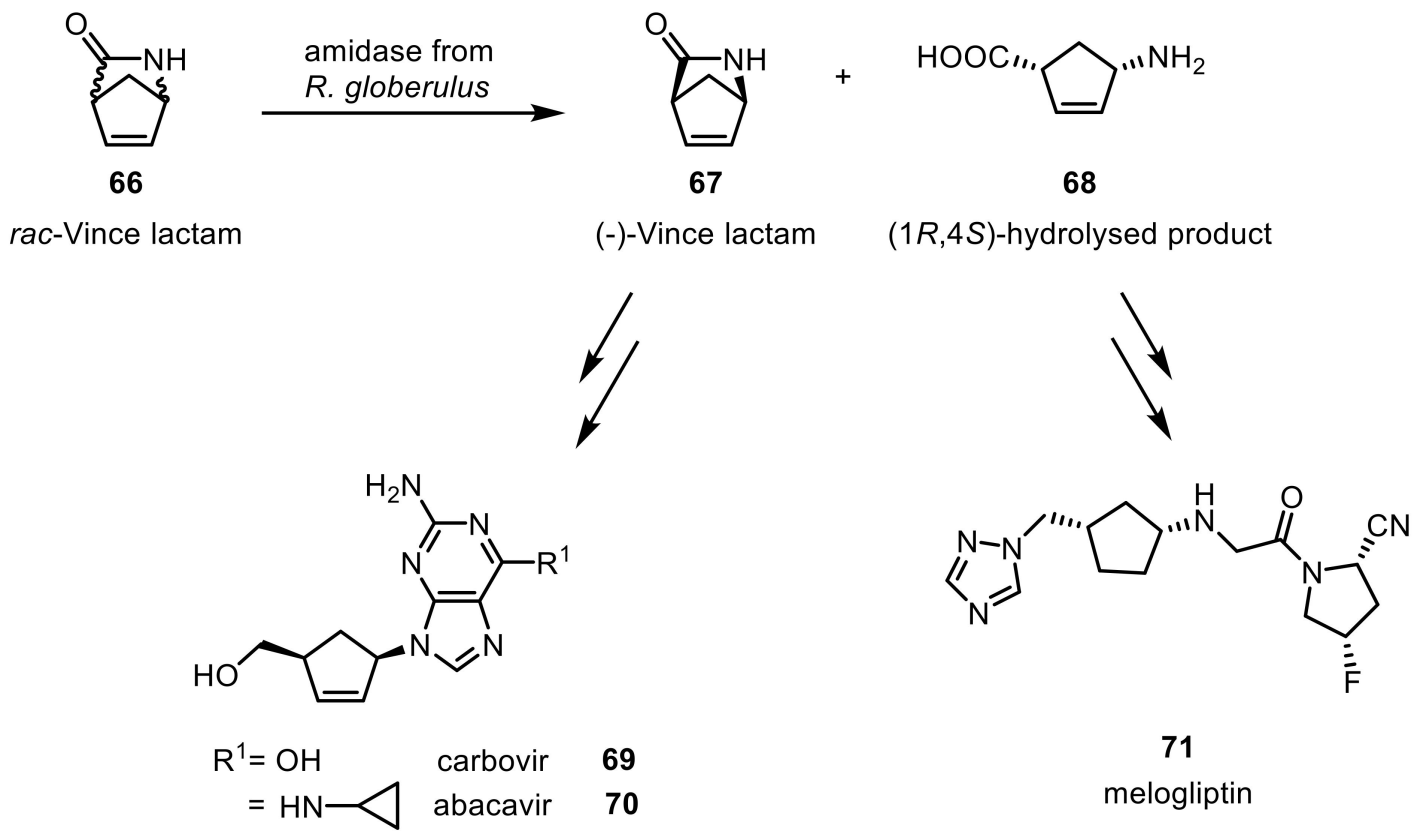
Kinetic resolution of Vince-lactam using an amidase from *R. globerulus* with subsequent follow-up chemistry of enantiopure products leading to antiviral compounds carbovir (**69**) and abacavir (**70**) and antidiabetic melogliptin (**71**) [180].

Lactams, cyclic analogues of amides, are generally more stable and their hydrolysis therefore often requires harsher reaction conditions leading to the respective amino acids [167]. Nonetheless, certain amidases (i.e., lactamases from *Rhodococcus*) were effectively used in the hydrolysis reaction. The hydrolysis of Vince-lactams (**66**) (2-azabicyclo[2.2.1]hept-5-en-3-one) and their derivatives have received increased attention due to the fact that the two resulting products are valuable synthons in the pharmaceutical industry for the manufacture of antiviral agents carbovir (**69**) and abacavir (**70**) or for the antidiabetic melogliptin (**71**) [178]. *R. equi* NCBI 40312 showed activity against Vince-lactam [179], and an amidase from *R. globerulus* presented excellent enantioselectivities for the obtained amino acid products (Scheme 20) [180].

Strains *R*. sp Oct1 and *R*. sp U224 were recently shown to act on various ω-lactams with the enzyme present in *R*. sp Oct1 having a more relaxed substrate scope, while the enzyme from *R*. sp U224 only accepted ω-octo- and ω-laurolactam [181,182].

### 3.4. Nitrilase

While all of the above-mentioned enzymes from *Rhodococcus* present in the aldoxime-nitrile pathway have been intensively studied and applied in many different processes and reactions, nitrilases (EC 3.5.5.1, or nitrile aminohydrolase) from *Rhodococcus* are less prominent and other microorganisms (*Synechocystis*, *Alcaligenes*, *Pseudomonas*, etc.) clearly dominate this area of research. However, a small number of reactions have been described using *Rhodococcus* whole-cells or purified enzymes.

Bioconversions of acrylonitrile with *R. rhodochrous* J1 yielded acrylic acid with exceptional yields of 390 g/L [183], which were even further improved with a mutant strain of *R. rhodochrous* tg1-A6 leading to 414.5 g/L [184].

The production of ammonium acrylate by two *Rhodococcus* isolates was further investigated and compared [185]. While *R. erythropolis* 3843 expressed a nitrile hydratase/amidase system, *R. ruber* NCIMB 40757 expressed a nitrilase. In this comparative study, the single-enzyme nitrilase-system was superior to the two-enzyme carboxylic acid production due to favorable reaction kinetics and because the NHase and amidase do not share the same temperature optimum.

*R. rhodochrous* PA-34 was shown to hydrolyze α-aminonitriles to obtain optically active **l**-amino acids (amongst others **l**-leucine, **l**-valine and **l**-methionine) as well as **d**-alanine [186]. Furthermore, strains *R. rhodochrous* J1 and R. sp. NDB 1165 were used to generate nicotinic acid, also called niacin, starting from 3-cyanopyridine with yields of 172 and 196.8 g/L, respectively [187,188]. Nicotinic acid is, next to nicotinamide, a form of vitamin B3 and its synthesis is therefore commercially interesting.

It is worth mentioning that even though only a small number of nitrilase-catalyzed reactions with *Rhodococcus* have been described, this nitrile-degrading enzyme class in general is well-studied and applied in a multitude of reactions, which can be followed-up on in several comprehensive reviews and books [114,189,190,191].

## 4. Hydrolase Activity in *Rhodococcus*

### 4.1. Epoxide Hydrolase (EC 3.3.2.x)

Epoxide hydrolases (EHs) catalyze the ring-opening reaction of oxirane rings, leading to vicinal diols and enantiopure epoxides as the final products. Most valuable are EHs that either show high enantioselectivities or a low enantiopreference with high levels of enantioconvergence. Many studies have focused on the asymmetric hydrolysis of geminal (2,2-disubstituted) oxiranes using *Rhododoccus* as a biocatalyst, whereby all investigated EHs hydrolyzed the (*S*)-enantiomer forming an (*S*)-diol and leaving the (*R*)-epoxide behind [192]. The investigated substrate scope includes linear alkyl-, alkenyl-, alkynyl- or benzylsubstituted epoxides. While *R. ruber* DSM 43338 acted on 2-methyl-2-(aryl)alkoxyoxiranes [193], *R*. sp. NCIMB 11216 was shown to convert (±)-2-methyl-2-alkyl-epoxides [194]. Oxiranes bearing an alkene or alkyne function were particularly well transformed using the same strain [195]. Additionally, two *Rhodococcus* strains isolated from marine sediments were used in the hydrolysis of styrene oxide and its chlorinated derivatives [196].

*R. erythropolis* DCL14 bears an epoxide hydrolase that holds a particular position within the family of EHs. While most of the described EHs belong to the α,β-hydrolase fold superfamily, the so-called ‘limonene-1,2-epoxide hydrolase’ (LEH) shows a 3-dimensional structure that is dissimilar to the others [197]. Consequently, it follows a different reaction mechanism, which was proposed as a single step ‘push–pull’ mechanism rather than a multiple-step mechanism described for members of the α,β-hydrolase fold superfamily [192]. Initial investigations showed a narrow substrate range for the wild-type enzyme. Only the natural substrate limonene-1,2-epoxide was converted with excellent stereoselectivity while several alicyclic and few 2-methyl-1,2-epoxides showed only reasonable stereoselectivities [198,199]. With an iterative saturation mutagenesis strategy, three LEH variants were developed with an expanded substrate range and opposite stereoselectivities, making this enzyme more attractive for future applications [200].

**Scheme 21:**
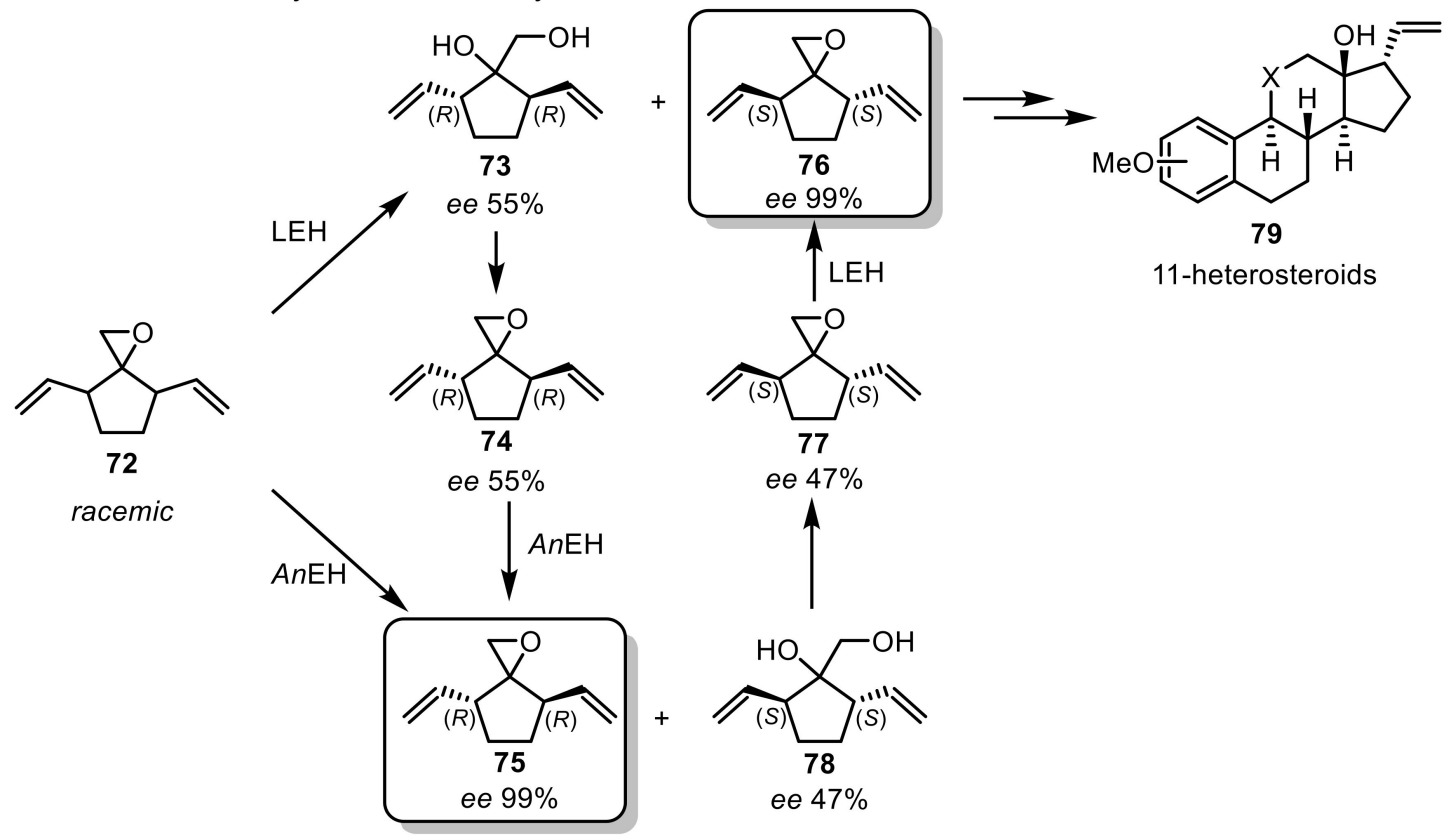
Optimized two-enzyme system for hydrolytic kinetic resolution of trans-spiroepoxide (**72**) using *An*EH and LEH. Enantiopure spiroepoxides (**75**,**76**) can be used in the synthesis of enantiopure 11-heterosteroids (**79**) [201].

A preparative hydrolytic kinetic resolution of *rac*-*trans*-spiroepoxides was first achieved with EHs from *Aspergillus niger* (*An*EH) and wild-type LEH from *R. erythropolis* DCL14. In the kinetic resolution of *rac*-*trans*-spiroepoxides (**72**), the two employed EHs exhibited opposite enantioselectivities, and by applying both enzymes in a combined two-step process, both enantiomers (**75**,**76**) were generated with excellent enantiomeric excess values and in high yields: the racemic spiroepoxide (**72**) was, for example, first hydrolyzed by LEH leading to the (*R*),(*R*)-diol (**73**) and (*S*),(*S*)-epoxide (**76**). Through a chemical recyclization the (*R*),(*R*)-diol (**73**) was transformed into (*R*),(*R*)-epoxide (**74**), keeping the moderate enantiopurity. The spiroepoxide (**74**) was then subsequently resolved by the enantiocomplementary EH from *A. niger*. Enantiopure spiroepoxides are seen as valuable synthons in the synthesis of 11-heterosteroids (Scheme 21) [201].

The asymmetric hydrolysis of (±)-2,3-*cis*- and *trans*-disubstituted oxiranes was performed by *Rhodococcus ruber* DSM 43338 with excellent enantioselectivities reaching 95% and 86% *ee*, respectively [202].

Next to geminal oxiranes, tri-substituted epoxides were also successfully converted. In an enantioconvergent fashion, whole-cells of *R. ruber* SM 1789 catalyzed the hydrolysis of trialkyl-oxiranes achieving high yields of the respective (*R*)-configured vicinal diols with high *ee* values [203]. Expanding the epoxide hydrolase system to substrates bearing olefinic side-chains enabled the straightforward asymmetric synthesis of natural compounds myrcenediol and 7,7-dimethyl-6,8-dioxabicyclo[3.2.1]octane, which is known to be a volatile contributor to the aroma of beer in a chemoenzymatic reaction procedure [204]. In another enantioconvergent asymmetric synthesis using an EH from *Rhodococcus*, it was possible to synthesize the monoterpenoid coumarin (*R*)-(+)-marmin with 95% *ee* under anaerobic reaction conditions [205].

**Scheme 22:**
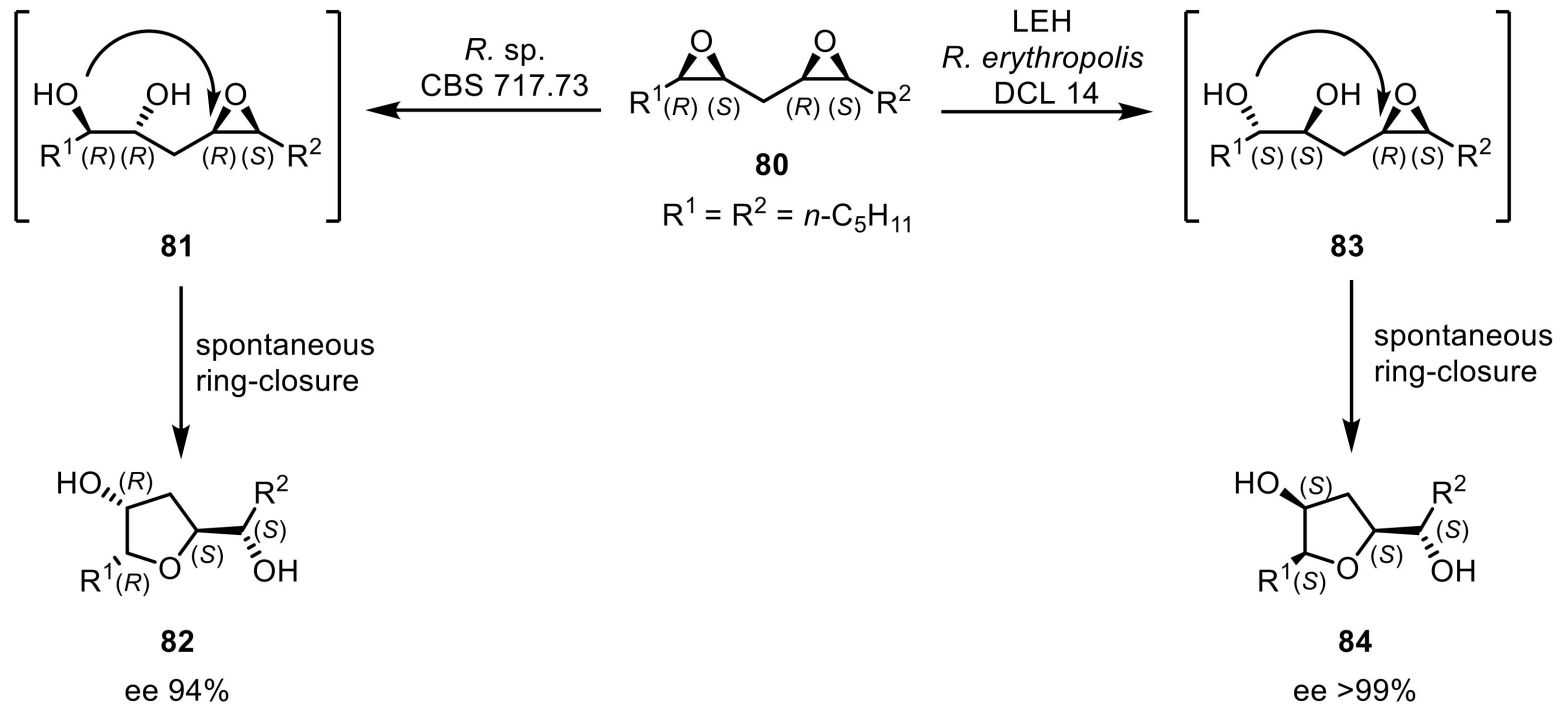
Stereochemical course of hydrolysis/cyclisation reaction catalyzed by two *Rhodococcus* representatives.

The biocatalytic desymmetrization of *meso*-epoxides with EHs was the focus of other studies: an EH from *R. ruber* ML-0004 was expressed in *E. coli* that showed a selective activity toward *cis*-epoxysuccinate producing enantiopure **l**-(+)-tartaric acid [206]. An enzyme-triggered hydrolysis-cyclisation reaction was observed when transforming methylene-interrupted meso-*bis*-epoxide (**80**) (6,7:9,10-*bis*(epoxy)pentadecane) with whole-cells of *R*. sp. CBS717.73 (Scheme 22). With this reaction, in total, four stereocenters were established in the obtained THF-derived product (**82**) [207]. Expansion of this study showed that four different THF-derivatives (representative **82**,**84**) each with excellent *ee* and *de* values were achieved by using EHs from multiple organisms (e.g., *Rhodococcus* species, *Solanum tuberosum*, *Aspergillus niger*, *Mycobacterium tuberculosis*). The stereospecificities of the enzymes thereby determined the final stereoselectivities of the products while the cyclization reaction follows Baldwin’s rule without the operation of a cyclase [208].

The three aforementioned variants of LEH obtained by an iterative saturation mutagenesis approach were applied in the hydrolytic desymmetrization of several *meso*-epoxides and *cis*-1,2-homodesubstituted *meso*-epoxides forming both the (*R*,*R*)- and (*S*,*S*)-diols with very good *ee* values [200].

The resolution of *rac*-2-methylglycidyl benzyl ether, a versatile building block, was achieved through a whole-cell bioconversion of *R. ruber* SM 1789 yielding (*R*)-vicinal diol [209]. Following this study, several other strains were tested on the same substrate. While most *Rhodococcus* strains showed (*S*)-selectivity and acted with the retention of the stereoselectivity, the limonene epoxide hydrolase from *R. erythropolis* showed an inversion of the substrate, leading to a homochiral mixture [210]. *R. ruber* CBS 717.73 catalyzed the hydrolysis of 2-benzyloxymethyl-2-methyloxirane, another protected epoxy-alcohol, and was applied in a chemoenzymatic route to produce (*S*)-chromanemethanol, an important building block in Vitamin E synthesis, in 26% overall yield and 99% *ee* [211].

**Scheme 23:**
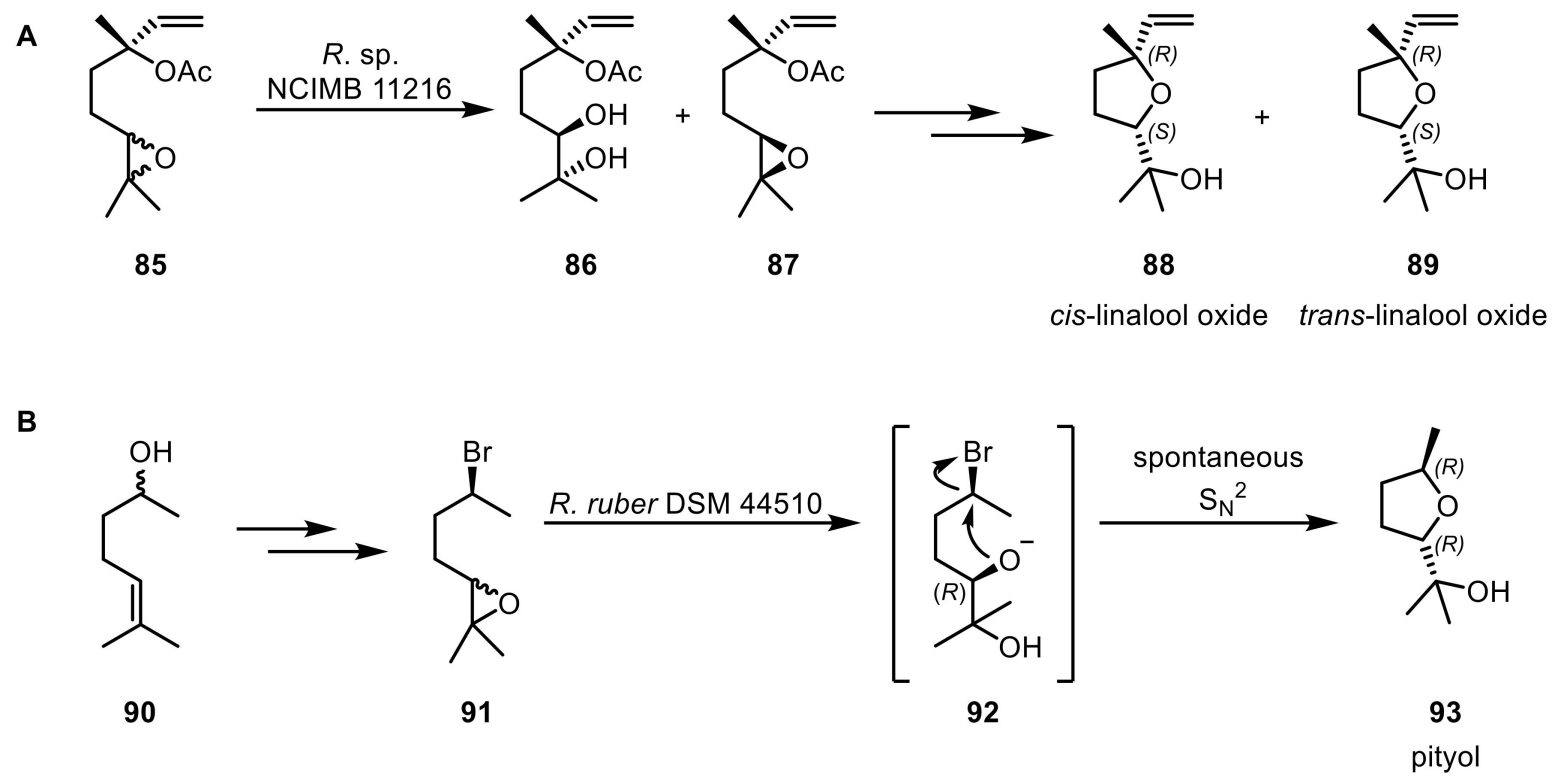
Natural product synthesis of (**A**) *cis*-(88) and *trans*-linalool oxides (89) and (**B**) pityol (93) using *Rhodococcus* whole-cell biocatalysts.

As portrayed, epoxide hydrolases from *Rhodococcus* are biocatalysts forming valuable synthons that can be used in a multitude of follow-up reactions. Next to the described application potential, *Rhodococcus* was also utilized in the production of other natural products such as linalool oxides (**88**,**89**) or pityol (**93**). *Cis*-(**88**) and *trans*-linalool (**89**) oxides were obtained via a chemo-enzymatic route starting from (3*RS*,6*R*)-2,3-epoxylinalyl acetate (**85**) with the *R*. sp NCIMB 11216 mediated resolution of the diastereomeric mixture of the starting compound as a key step (Scheme 23A) [212]. (2*R*,5*R*)-pityol (**93**), a bark beetle pheromone, was synthesized starting from (±)-sulcatol (**90**) via an lipase-catalyzed deracemization and a subsequent EH-catalyzed diastereoconvergent hydrolysis of a haloalkyl oxirane (**91**) (Scheme 23B) [213].

### 4.2. Esterase Activity

**Scheme 24:**
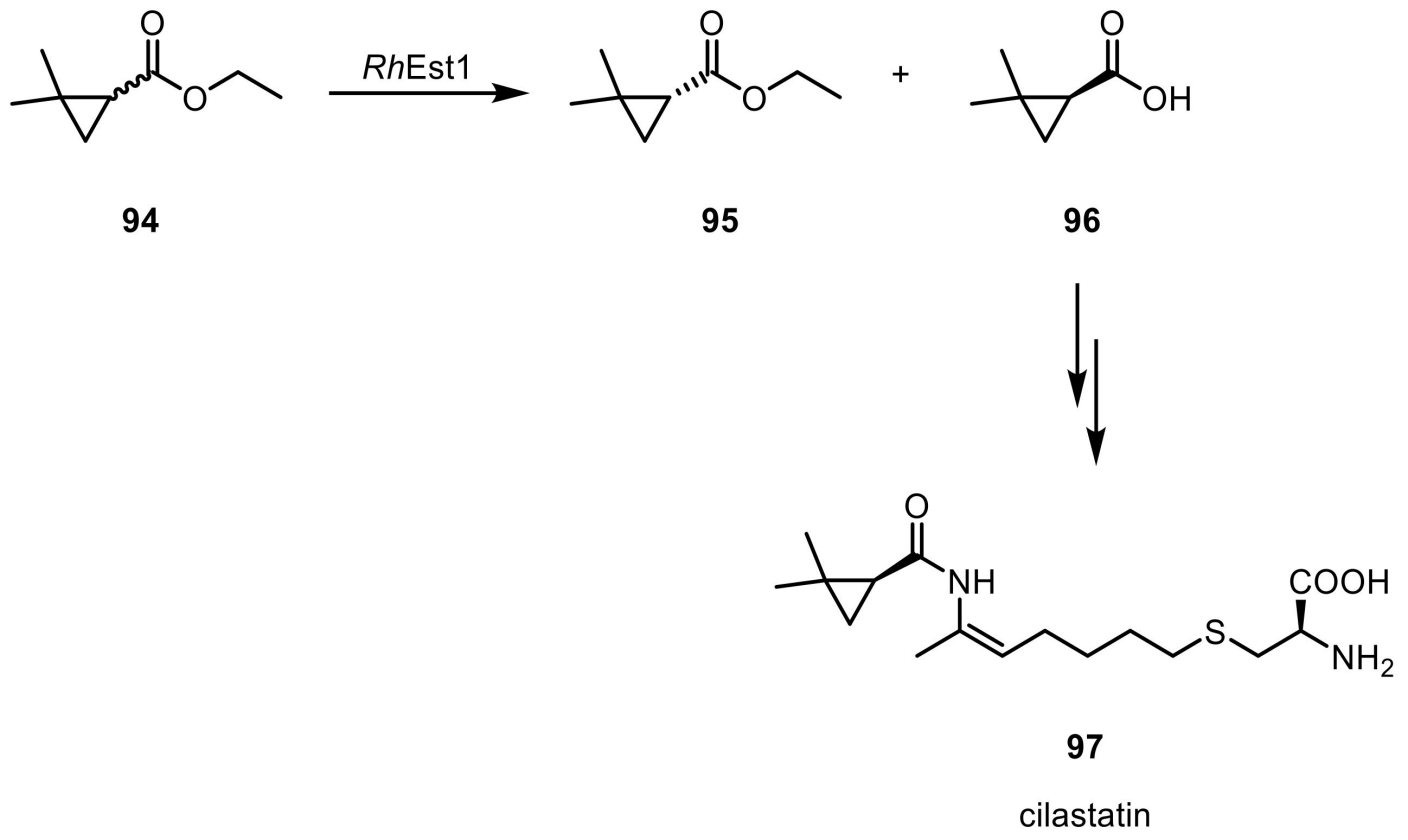
Enantioselective resolution of a racemic mixture of DMCPCM (**94**) to yield (*S*)-DMCPCA (**96**)—a precursor for the drug cilastatin (**97**) [214].

Few reports have concentrated on the application of other hydrolases from *Rhodococcus*. Amongst them, an esterase from *R*. sp. ECU1013 (RhEst1) was shown to hydrolyze *rac*-ethyl-2,2-dimethyl-*c*-propanecarboxylate (**94**, rac-DMCPCM) to give (*S*)-(+)-2,2-dimethyl-c-propylcarboxylic acid (**96**, (*S*)-DMCPCA)—a valuable precursor in the synthesis of the drug cilastatin (**97**) (Scheme 24) [214,215]. This shows an alternative route to cilastatin compared to the previously mentioned nitrile hydratase-catalyzed process starting with 2,2-dimethyl-c-propanecarbonitrile [136].

The production of both (*S*)- and (*R*)-linalool was achieved with two (partially) purified enzymes ((*S*)- and (*R*)-linalyl acetate hydrolase, respectively) from *R. ruber* DSM 43338 via the hydrolysis of the corresponding acetate esters [216]. An urethane hydrolase from *R. equi* TB-60 hydrolyzed a diverse range of compounds including anilides, amides, and esters such as toluene-2,4-dicarbamic acid butyl ester (TDCB) [217].

## 5. Hydratase Activity

Hydratases catalyze the addition of water to (un)-activated double bonds to form valuable enantiopure alcohols. It can be stated that nitrile hydratases from *Rhodococcus* have been profoundly investigated and were the center of research regarding hydratase activity. However, several *Rhodococcus* strains have shown an intriguing hydration potential toward other diverse compounds and their development is on the rise.

### 5.1. Oleate Hydratase

**Figure 1:**
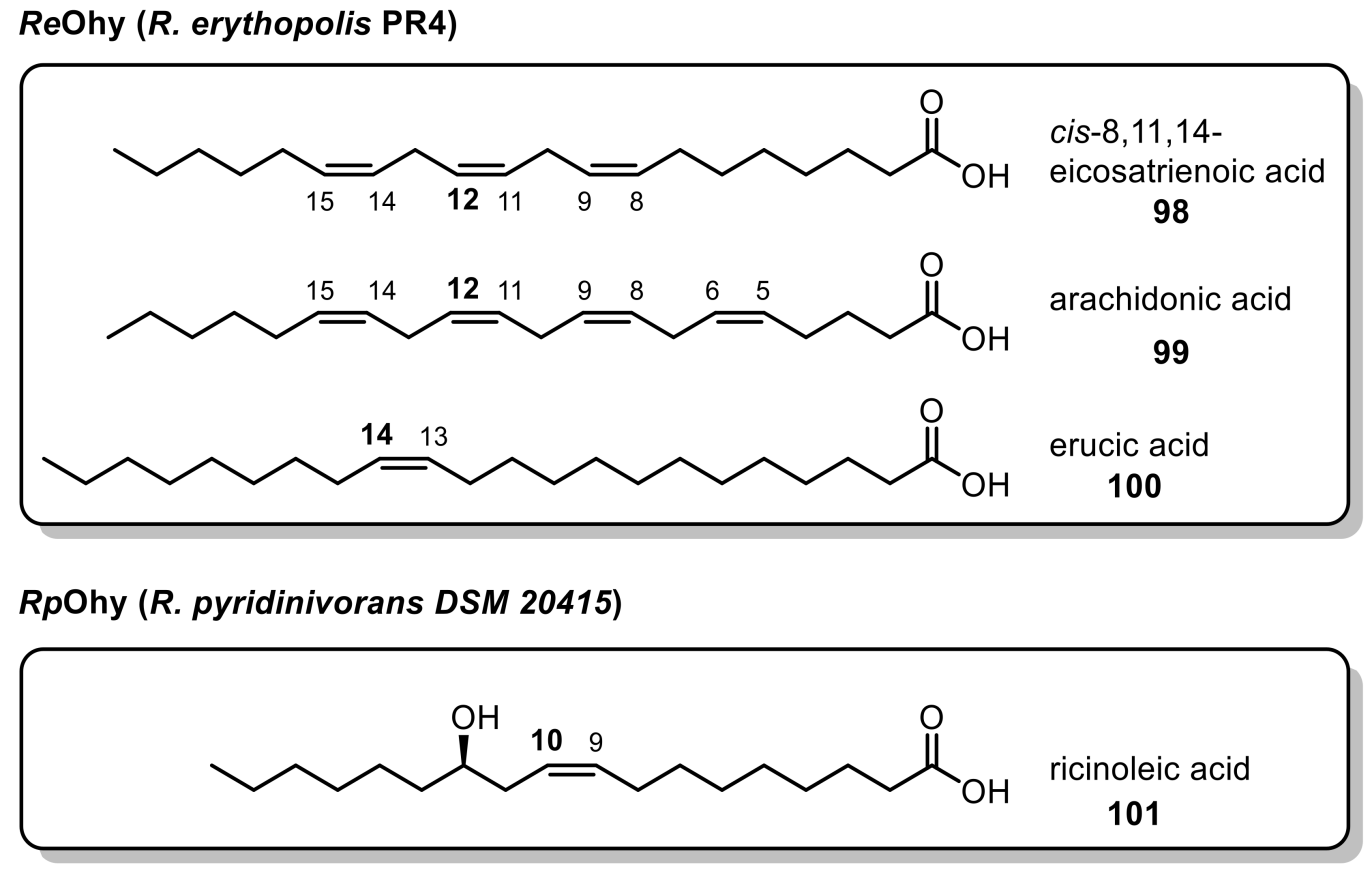
Exemplary display of complementary substrate scope of two Ohy from *Rhodococcus*. The bold number indicates the location of the hydroxy-group in the final hydrated product.

Recently, a novel oleate hydratase (Ohy, EC 4.2.1.53) was isolated from *R. erythropolis* CCM 2595 and characterized [218]. Structural analysis showed that this Ohy differed structurally from the previously described bacterial Ohys. This enzyme was shown to be an active monomer while all other characterized Ohys showed dimeric structures instead [219,220,221]. The purified Ohy was shown to accept a small range of unsaturated fatty acids, adding water exclusively in the 10-position while more complex lipids were not converted [218]. *In-silico* analysis of the occurrence and phylogenetic relationship of annotated oleate hydratases in 43 *Rhodococcus* strains revealed that distinct *Rhodococcus* clades showed Ohy potential, thereby discovering 19 novel oleate hydratases [222]. Two representatives, Ohys from *R. erythropolis* PR4 and *R. pyridinivorans* DSM 20415, sharing a sequence similarity and identity of 46% and 30%, respectively, were heterologously expressed and tested on (multiple) unsaturated fatty acids. While both Ohys converted smaller fatty acids in the same manner, they showed a complementary substrate scope toward sterically demanding and longer fatty acids (Figure 1).

A recently filed patent involves the Ohy-catalyzed conversion of free fatty acids such as oleic acid derived from the renewable feedstock of bio-based oils [223]. This is one example of the successful use of a novel Ohy from *Rhodococcus* that further improves the potential application of *Rhodococcus* as a useful biocatalyst.

### 5.2. Michael Hydratase

Whole-cells of *R. rhodochrous* ATCC 17896 were shown to catalyze a so-called Michael addition of water to α,β-unsaturated carbonyl compound (*E*)-4-hydroxy-3-methylbut-2-enoic acid and its respective ethyl-derivative [224]. Upon water addition, an internal nucleophilic attack leads to the formation of useful synthon (*R*)-4-hydroxy-4-methyldihydrofuran-2(3H)-one. The stereochemical course of the reaction was investigated, showing that the water-addition takes place in *syn-*fashion [225,226]. Interestingly, this hydratase demonstrated unusual behavior as it requires oxygen for higher activities, but labeling studies with D_2_O and ^18^O_2_ proved a true water addition instead of an oxidative process [226]. Unfortunately, this membrane-associated enzyme has not been isolated and the coding gene remains elusive up to this point.

## 6. *Rhodococcus* Acting on Sulfur-Containing Compounds

### 6.1. Sulfatase

**Scheme 25:**
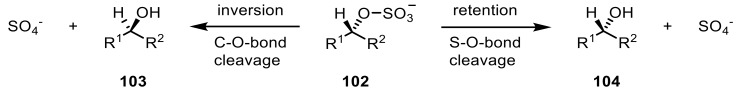
Retention or conversion during enzymatic sulfate ester hydrolysis catalyzed by sulfatase.

Sulfatases (EC 3.1.6.x) are a group of enzymes that catalyze the hydrolytic cleavage of sulfate esters by releasing inorganic sulfate and the corresponding alcohols [227]. Fascinatingly, sulfatases can achieve an enantio-convergent transformation of racemic sulfate esters (**102**) into only one stereoisomeric secondary alcohol yielding up to 100% yield theoretically, thereby surpassing traditional kinetic resolution processes. Sulfatases thereby determine not only the enantioselectivity, but also the stereoselectivity with respect to the retention (**104**) or inversion (**103**) of the configuration (Scheme 25) [228].

The hydrolysis of an alkyl-sulfate through the breakage of the S-O-bond by nucleophilic attack leads to a retention while the breakage of the C–O-bond on the other hand leads to an inversion of the configuration at the carbon atom bearing the center of chirality [228]. *R. ruber* DSM 44541 was shown to have a secondary alkyl sulfatase, called RS2, which acts through strict inversion. Methyl- and ethyl-(alkyl)sulfate esters with a varying chain-length were shown to be hydrolyzed [229,230]. The addition of Fe^3+^-ions led to an increase in activity, which, however, was not suitable for preparative scale reactions [231,232].

While most of the investigations concentrated on secondary alkyl-sulfates, few reports about cyclic sulfates have come forward. Growing cells of *R*. sp. CCZU10-1, however, were used to transform four cyclic sulfates: 1,3-propanediol cyclic sulfate (1,3-PDS), 1,2-propanediol cyclic sulfate (1,2-PDS), ethylene sulfate, and glycol sulfate [227]. All four cyclic sulfates were hydrolyzed, which makes this the first time that a *Rhodococcus* species acted on sulfate or sulfite, thereby generating diols.

### 6.2. Sulfide Monooxygenase

Chiral sulfoxides are key synthons in chiral drug production and are used as versatile auxiliary compounds as chiral ligands or catalysts. The asymmetric oxidation of prochiral sulfides catalyzed by whole-cell biocatalysts is superior to classical chemical routes [233]. Phenyl methyl sulfide (PMS) was oxidized into (*S*)-phenyl methyl sulfoxide (PMSO) by whole-cells of *R*. sp. ECU0066 [234,235] and *R*. sp. CCZU10-1 [233]. Both studies showed the expansion of the substrate scope to a small set of PMS derivatives. A sulfide/sulfoxide flavin-dependent monooxygenase from *R*. sp. IGTS8 was produced in *E. coli* and investigated in detail [236]. It was shown that the enzyme oxidizes DBT in two consecutive steps, yielding DBT sulfone.

## 7. Conclusions

Enzymes present in *Rhodococcus* strains cover a broad field of diverse reactions ranging from redox-reactions and hydrolysis reactions of epoxides or esters to the hydration of fatty acids or Michael acceptors. The most prominent and best exploited enzymes in *Rhodococcus* are mostly the enzymes present in the aldoxime-nitrile pathway, with the nitrile hydratase being the prime example of a successful biocatalyst being run on a multi-ton industrial scale process. On the other hand, enzymes that have only recently been discovered like aldoxime dehydratase or enzymes acting on sulfur-containing compounds have the potential for further development as the industrial biocatalysts of the future.

Genome mining as well as the isolation of more *Rhodococcus* strains especially from rare sites opens up the chance to identify new enzymes with interesting properties such as an extraordinarily broad substrate scope, higher enantio- or stereoselectivities for defined reactions, better overall stabilities at extreme temperatures, or unusual reaction media. Additionally, the engineering of known enzymes to enhance their function also increases the number of possible chemical reactions. One such example is the evolution of amine dehydrogenase from amino acid dehydrogenase, which led to a straightforward synthesis of (*R*)-selective amines, which had been a major challenge in the past.

Few reactions have been carried out with purified enzymes while the majority of reactions shown were catalyzed by either *Rhodococcus* whole-cells (wild-type or mutants) or *E. coli* whole-cell reactions with heterologously expressed enzymes from *Rhodococcus*. It was shown that enzymes from *Rhodococcus* are valuable resources in the design of novel biocascades, which will attract only more attention in the near future. Here, the combination of enzymes in well-established expression hosts is theoretically boundless given that the present enzymes share reaction condition ranges (e.g., pH, temperature, solvent) and act on the same desired compounds.

In summary, the genus *Rhodococcus* truly deserves to be termed as a ‘biocatalytic powerhouse’: its enzymatic diversity and overall robustness make these microorganisms one of the key players in many areas of biocatalysis and will continue to do so.

## References

[1] Alvarez H.M. (2019). Biology of Rhodococcus.

[2] Larkin M.J., Kulakov L.A., Allen C.C.R. (2006). Biodegradation by Members of the Genus *Rhodococcus*: Biochemistry, Physiology, and Genetic Adaptation. Adv. Appl. Microbiol..

[3] Zampolli J., Zeaiter Z., Di Canito A., Di Gennaro P. (2019). Genome analysis and -omics approaches provide new insights into the biodegradation potential of *Rhodococcus*. Appl. Microbiol. Biotechnol..

[4] Abdelraheem E.M.M., Busch H., Hanefeld U., Tonin F. (2019). Biocatalysis explained: From Pharmaceutical to Bulk Chemical Production. React. Chem. Eng..

[5] Gröger H., Asano Y., Bornscheuer U.T., Ogawa J. (2012). Development of biocatalytic processes in Japan and Germany: From research synergies to industrial applications. Chem. Asian J..

[6] Ogawa J., Shimizu S. (2002). Industrial microbial enzymes: Their discovery by screening and use in large-scale production of useful chemicals in Japan. Curr. Opin. Biotechnol..

[7] Kim D., Choi K.Y., Yoo M., Zylstra G.J., Kim E. (2018). Biotechnological potential of *Rhodococcus* biodegradative pathways. J. Microbiol. Biotechnol..

[8] Larkin M.J., Kulakov L.A., Allen C.C.R. (2005). Biodegradation and *Rhodococcus*—Masters of catabolic versatility. Curr. Opin. Biotechnol..

[9] Liang Y., Jiao S., Wang M., Yu H., Shen Z. (2019). Overexpression of epoxide hydrolase in *Rhodococcus ruber* with high robustness for the synthesis of chiral epichlorohydrin. Process Biochem..

[10] Van Der Geize R., Dijkhuizen L. (2004). Harnessing the catabolic diversity of Rhodococci for environmental and biotechnological applications. Curr. Opin. Microbiol..

[11] Kim D., Kim Y.S., Kim S.K., Kim S.W., Zylstra G.J., Kim Y.M., Kim E. (2002). Monocyclic aromatic hydrocarbon degradation by *Rhodococcus* sp. strain DK17. Appl. Environ. Microbiol..

[12] Kim S.H., Han H.Y., Lee Y.J., Kim C.W., Yang J.W. (2010). Effect of electrokinetic remediation on indigenous microbial activity and community within diesel contaminated soil. Sci. Total Environ..

[13] Ahmad M., Roberts J.N., Hardiman E.M., Singh R., Eltis L.D., Bugg T.D.H. (2011). Identification of DypB from *Rhodococcus jostii* RHA1 as a lignin peroxidase. Biochemistry.

[14] Mycroft Z., Gomis M., Mines P., Law P., Bugg T.D.H. (2015). Biocatalytic conversion of lignin to aromatic dicarboxylic acids in *Rhodococcus jostii* RHA1 by re-routing aromatic degradation pathways. Green Chem..

[15] Wei Z., Wilkinson R.C., Rashid G.M.M., Brown D., Fülöp V., Bugg T.D.H. (2019). Characterization of Thiamine Diphosphate-Dependent 4-Hydroxybenzoylformate Decarboxylase Enzymes from *Rhodococcus jostii* RHA1 and *Pseudomonas fluorescens* Pf-5 Involved in Degradation of Aryl C-2 Lignin Degradation Fragments. Biochemistry.

[16] Masai E., Yamada A., Healy J.M., Hatta T., Kimbara K., Fukuda M., Yano K. (1995). Characterization of biphenyl catabolic genes of gram-positive polychlorinated biphenyl degrader *Rhodococcus* sp. strain RHA1. Appl. Environ. Microbiol..

[17] Khairy H., Wübbeler J.H., Steinbüchel A. (2015). Biodegradation of the Organic Disulfide 4,4′-Dithiodibutyric Acid by *Rhodococcus* spp.. Appl. Environ. Microbiol..

[18] Anteneh Y.S., Franco C.M.M. (2019). Whole cell actinobacteria as biocatalysts. Front. Microbiol..

[19] Hollmann F., Faber K., Fessner W.-D., Turner N.J. (2015). Oxidation using Dehydrogenases. Biocatalysis in Organic Synthesis 3.

[20] Schenkels P., Duine J.A. (2000). Nicotinoprotein (NADH-containing) alcohol dehydrogenase from *Rhodococcus erythropolis* DSM 1069: An efficient catalyst for coenzyme-independent oxidation of a broad spectrum of alcohols and the interconversion of alcohols and aldehydes. Microbiology.

[21] Stampfer W., Kosjek B., Moitzi C., Kroutil W., Faber K. (2002). Biocatalytic asymmetric hydrogen transfer. Angew. Chem. Int. Ed..

[22] Nikodinovic J., Dinges J.M., Bergmeier S.C., McMills M.C., Wright D.L., Priestley N.D. (2006). Resolution of methyl nonactate by *Rhodococcus erythropolis* under aerobic and anaerobic conditions. Org. Lett..

[23] Voss C.V., Gruber C.C., Faber K., Knaus T., Macheroux P., Kroutil W. (2008). Orchestration of concurrent oxidation and reduction cycles for stereoinversion and deracemisation of sec-alcohols. J. Am. Chem. Soc..

[24] Voss C.V., Gruber C.C., Kroutil W. (2008). Deracemization of secondary alcohols through a concurrent tandem biocatalytic oxidation and reduction. Angew. Chem. Int. Ed..

[25] Wu S., Li Z. (2018). Whole-Cell Cascade Biotransformations for One-Pot Multistep Organic Synthesis. ChemCatChem.

[26] Müller C.A., Weingartner A.M., Dennig A., Ruff A.J., Gröger H., Schwaneberg U. (2016). A whole cell biocatalyst for double oxidation of cyclooctane. J. Ind. Microbiol. Biotechnol..

[27] Both P., Busch H., Kelly P.P., Mutti F.G., Turner N.J., Flitsch S.L. (2016). Whole-Cell Biocatalysts for Stereoselective C-H Amination Reactions. Angew. Chem. Int. Ed..

[28] Biermann M., Gruß H., Hummel W., Gröger H. (2016). Guerbet Alcohols: From Processes under Harsh Conditions to Synthesis at Room Temperature under Ambient Pressure. ChemCatChem.

[29] Stankevičiūtė J., Kutanovas S., Rutkienė R., Tauraitė D., Striela R., Meškys R. (2015). Ketoreductase TpdE from *Rhodococcus jostii* TMP1: Characterization and application in the synthesis of chiral alcohols. PeerJ.

[30] Gröger H., Hummel W., Rollmann C., Chamouleau F., Hüsken H., Werner H., Wunderlich C., Abokitse K., Drauz K., Buchholz S. (2004). Preparative asymmetric reduction of ketones in a biphasic medium with an (*S*)-alcohol dehydrogenase under in situ-cofactor-recycling with a formate dehydrogenase. Tetrahedron.

[31] Pollard D., Truppo M., Pollard J., Chen C.-Y., Moore J. (2006). Effective synthesis of (*S*)-3,5-bistrifluoromethylphenyl ethanol by asymmetric enzymatic reduction. Tetrahedron Asymmetry.

[32] Liu Y.C., Wu Z.L. (2016). Switchable asymmetric bio-epoxidation of α,β-unsaturated ketones. Chem. Commun..

[33] Grau B.T., Devine P.N., DiMichele L.N., Kosjek B. (2007). Chemo- and enantioselective routes to chiral fluorinated hydroxyketones using ketoreductases. Org. Lett..

[34] Borzecka W., Lavandera I., Gotor V. (2013). Synthesis of enantiopure fluorohydrins using alcohol dehydrogenases at high substrate concentrations. J. Org. Chem..

[35] Busto E., Gotor-Fernández V., Gotor V. (2012). Asymmetric chemoenzymatic synthesis of ramatroban using lipases and oxidoreductases. J. Org. Chem..

[36] Busto E., Martínez-Montero L., Gotor V., Gotor-Fernández V. (2013). Chemoenzymatic asymmetric synthesis of serotonin receptor agonist (*R*)-frovatriptan. Eur. J. Org. Chem..

[37] Méndez-Sánchez D., Mourelle-Insua Á., Gotor-Fernández V., Lavandera I. (2019). Synthesis of α-Alkyl-β-Hydroxy Amides through Biocatalytic Dynamic Kinetic Resolution Employing Alcohol Dehydrogenases. Adv. Synth. Catal..

[38] Mangas-Sánchez J., Busto E., Gotor V., Gotor-Fernández V. (2013). One-pot synthesis of enantiopure 3,4-dihydroisocoumarins through dynamic reductive kinetic resolution processes. Org. Lett..

[39] Ying X., Zhang J., Wang C., Huang M., Ji Y., Cheng F., Yu M., Wang Z., Ying M. (2018). Characterization of a carbonyl reductase from *Rhodococcus erythropolis* WZ010 and its variant Y54F for asymmetric synthesis of (*S*)-*N*-Boc-3-hydroxypiperidine. Molecules.

[40] Ewing T.A., Fraaije M.W., van Berkel W.J.H., Faber K., Fessner W.-D., Turner N.J. (2015). Oxidation using Alcohol oxidases. Biocatalysis in Organic Synthesis 3.

[41] Jin J., Mazon H., Van Den Heuvel R.H.H., Janssen D.B., Fraaije M.W. (2007). Discovery of a eugenol oxidase from *Rhodococcus* sp. strain RHA1. FEBS J..

[42] Dieth S., Tritsch D., Biellmann J.-F. (1995). Resolution of allylic alcohols by cholesterol oxidase isolated from *Rhodococcus erythropolis*. Tetrahedron Lett..

[43] Wu K., Li W., Song J., Li T. (2015). Production, Purification, and Identification of Cholest-4-en-3-one Produced by Cholesterol Oxidase from *Rhodococcus* sp. in Aqueous/Organic Biphasic System. Biochem. Insights.

[44] Bertola M.A., Koger H.S., Phillips G.T., Marx A.F., Claassen V.P. (1987). A process for the preparation of (*R*)- and (*S*)-2,2-R1,R2-1,3-dioxolane-4-methanol.

[45] Liese A., Seelbach K., Buchholz A., Haberland J., Liese A., Seelbach K., Wandrey C. (2006). Processes. Industrial Biotransformations.

[46] Nolte J.C., Urlacher V.B., Faber K., Fessner W.-D., Turner N.J. (2015). Cytochrome P450 in the oxidation of alkenes. Biocatalysis in Organic Synthesis 3.

[47] Roper L., Grogan G., Goswami A., Stewart J.D. (2015). Biocatalysis for Organic Chemists: Hydroxylations. Organic Synthesis Using Biocatalysis.

[48] Roberts G.A., Celik A., Hunter D.J.B., Ost T.W.B., White J.H., Chapman S.K., Turner N.J., Flitsch S.L. (2003). A self-sufficient cytochrome p450 with a primary structural organization that includes a flavin domain and a [2Fe-2S] redox center. J. Biol. Chem..

[49] Roberts G.A., Grogan G., Greter A., Flitsch S.L., Turner N.J. (2002). Identification of a new class of cytochrome P450 from a *Rhodococcus* sp.. J. Bacteriol..

[50] O’Reilly E., Corbett M., Hussain S., Kelly P.P., Richardson D., Flitsch S.L., Turner N.J. (2013). Substrate promiscuity of cytochrome P450 RhF. Catal. Sci. Technol..

[51] Sabbadin F., Grogan G., Bruce N.C. (2010). LICRED: A versatile drop-in vector for rapid generation of redox-self-sufficient cytochromes P450. Methods Mol. Biol..

[52] De Gonzalo G., van Berkel W.J.H., Fraaije M.W., Faber K., Fessner W.-D., Turner N.J. (2015). Baeyer-Villiger Oxidation. Biocatalysis in Organic Synthesis 3.

[53] Brzostowicz P.C., Walters D.M., Thomas S.M., Nagarajan V., Rouvière P.E. (2003). mRNA differential display in a microbial enrichment culture: Simultaneous identification of three cyclohexanone monooxygenases from three species. Appl. Environ. Microbiol..

[54] Rudroff F., Rydz J., Ogink F.H., Fink M., Mihovilovic M.D. (2007). Comparing the stereoselective biooxidation of cyclobutanones by recombinant strains expressing bacterial baeyer—Villiger monooxygenases. Adv. Synth. Catal..

[55] Mihovilovic M.D., Snajdrova R., Grötzl B. (2006). Microbial Baeyer-Villiger oxidation of 4,4-disubstituted cyclohexan- and cyclohexenones by recombinant whole-cells expressing monooxygenases of bacterial origin. J. Mol. Catal. B Enzym..

[56] Snajdrova R., Braun I., Bach T., Mereiter K., Mihovilovic M.D. (2007). Biooxidation of bridged cycloketones using Baeyer-Villiger monooxygenases of various bacterial origin. J. Org. Chem..

[57] Rehdorf J., Mihovilovic M.D., Fraaije M.W., Bornscheuer U.T. (2010). Enzymatic synthesis of enantiomerically pure β-amino ketones, β-amino esters, and β-amino alcohols with Baeyer-Villiger monooxygenases. Chem. A Eur. J..

[58] Kostichka K., Thomas S.M., Gibson K.J., Nagarajan V., Cheng Q. (2001). Cloning and characterization of a gene cluster for cyclododecanone oxidation in *Rhodococcus ruber* SC1. J. Bacteriol..

[59] Fink M.J., Rudroff F., Mihovilovic M.D. (2011). Baeyer-Villiger monooxygenases in aroma compound synthesis. Bioorg. Med. Chem. Lett..

[60] Montersino S., van Berkel W.J.H. (2012). Functional annotation and characterization of 3-hydroxybenzoate 6-hydroxylase from *Rhodococcus jostii* RHA1. Biochim. Biophys. Acta Proteins Proteom..

[61] McLoed M.P., Warren R.L., Hsiao W.W.L., Araki N., Myhre M., Fernandes C., Miyazawa D., Wong W., Lillquist A.L., Wang D. (2006). The complete genome of *Rhodococcus* sp. RHA1 provides insights into a catabolic powerhouse. PNAS.

[62] Riebel A., Dudek H.M., De Gonzalo G., Stepniak P., Rychlewski L., Fraaije M.W. (2012). Expanding the set of rhodococcal Baeyer-Villiger monooxygenases by high-throughput cloning, expression and substrate screening. Appl. Microbiol. Biotechnol..

[63] Riebel A., De Gonzalo G., Fraaije M.W. (2013). Expanding the biocatalytic toolbox of flavoprotein monooxygenases from *Rhodococcus jostii* RHA1. J. Mol. Catal. B Enzym..

[64] Messiha H.L., Ahmed S.T., Karuppiah V., Suardíaz R., Ascue Avalos G.A., Fey N., Yeates S., Toogood H.S., Mulholland A.J., Scrutton N.S. (2018). Biocatalytic Routes to Lactone Monomers for Polymer Production. Biochemistry.

[65] Liu Y.-Y., Li C.-X., Xu J.-H., Zheng G.-W. (2019). Efficient Synthesis of Methyl 3-Acetoxypropionate by a newly identified Baeyer-Villiger Monooxygenase. Appl. Environ. Microbiol..

[66] Heine T., van Berkel W.J.H., Gassner G., van Pée K.H., Tischler D. (2018). Two-component FAD-dependent monooxygenases: Current knowledge and biotechnological opportunities. Biology.

[67] Paul C.E., Tischler D., Riedel A., Heine T., Itoh N., Hollmann F. (2015). Nonenzymatic Regeneration of Styrene Monooxygenase for Catalysis. ACS Catal..

[68] Riedel A., Heine T., Westphal A.H., Conrad C., Rathsack P., van Berkel W.J.H., Tischler D. (2015). Catalytic and hydrodynamic properties of styrene monooxygenases from *Rhodococcus opacus* 1CP are modulated by cofactor binding. AMB Express.

[69] Toda H., Imae R., Komio T., Itoh N. (2012). Expression and characterization of styrene monooxygenases of *Rhodococcus* sp. ST-5 and ST-10 for synthesizing enantiopure (*S*)-epoxides. Appl. Microbiol. Biotechnol..

[70] Toda H., Imae R., Itoh N. (2012). Efficient biocatalysis for the production of enantiopure (*S*)-epoxides using a styrene monooxygenase (SMO) and *Leifsonia* alcohol dehydrogenase (LSADH) system. Tetrahedron Asymmetry.

[71] Toda H., Imae R., Itoh N. (2014). Bioproduction of chiral epoxyalkanes using styrene monooxygenase from *Rhodococcus* sp. ST-10 (*Rh*SMO). Adv. Synth. Catal..

[72] Toda H., Ohuchi T., Imae R., Itoh N. (2015). Microbial production of aliphatic (*S*)-epoxyalkanes by using *Rhodococcus* sp. strain ST-10 styrene monooxygenase expressed in organic-solvent-tolerant *Kocuria rhizophila* DC2201. Appl. Environ. Microbiol..

[73] Tischler D., Eulberg D., Lakner S., Kaschabek S.R., Van Berkel W.J.H., Schlömann M. (2009). Identification of a novel self-sufficient styrene monooxygenase from *Rhodococcus opacus* 1CP. J. Bacteriol..

[74] Tischler D., Kermer R., Gröning J.A.D., Kaschabek S.R., Van Berkel W.J.H., Schlömann M. (2010). StyA1 and StyA2B from *Rhodococcus opacus* 1CP: A multifunctional styrene monooxygenase system. J. Bacteriol..

[75] Heine T., Grossmann C., Hofmann S., Tischler D. (2019). Indigoid dyes by group E monooxygenases: Mechanism and biocatalysis. Biol. Chem..

[76] Gakhar L., Malik Z.A., Allen C.C.R., Lipscomb D.A., Larkin M.J., Ramaswamy S. (2005). Structure and increased thermostability of *Rhodococcus* sp. naphthalene 1,2-dioxygenase. J. Bacteriol..

[77] Kim D., Yoo M., Choi K.Y., Kang B.S., Kim E. (2013). Characterization and engineering of an *o*-xylene dioxygenase for biocatalytic applications. Bioresour. Technol..

[78] Kim D., Choi K.Y., Yoo M., Choi J.N., Lee C.H., Zylstra G.J., Kang B.S., Kim E. (2010). Benzylic and aryl hydroxylations of *m*-xylene by *o*-xylene dioxygenase from *Rhodococcus* sp. strain DK17. Appl. Microbiol. Biotechnol..

[79] Kim D., Kim Y.S., Jung J.W., Zylstra G.J., Kim Y.M., Kim S.K., Kim E. (2003). Regioselective oxidation of xylene isomers by *Rhodococcus* sp. strain DK17. FEMS Microbiol. Lett..

[80] Kim D., Lee J.S., Choi K.Y., Kim Y.S., Choi J.N., Kim S.K., Chae J.C., Zylstra G.J., Lee C.H., Kim E. (2007). Effect of functional groups on the regioselectivity of a novel *o*-xylene dioxygenase from *Rhodococcus* sp. strain DK17. Enzym. Microb. Technol..

[81] Kim D., Yoo M., Choi K.Y., Kang B.S., Kim T.K., Hong S.G., Zylstra G.J., Kim E. (2011). Differential degradation of bicyclics with aromatic and alicyclic rings by *Rhodococcus* sp. strain DK17. Appl. Environ. Microbiol..

[82] Kim D., Lee C.H., Choi J.N., Choi K.Y., Zylstra G.J., Kim E. (2010). Aromatic hydroxylation of indan by *o*-xylene-degrading *Rhodococcus* sp. strain DK17. Appl. Environ. Microbiol..

[83] Chartrain M., Jackey B., Taylor C., Sandford V., Gbewonyo K., Lister L., Dimichele L., Hirsch C., Heimbuch B., Maxwell C. (1998). Bioconversion of indene to *cis* (1*S*,2*R*) indandiol and *trans* (1*R*,2*R*) indandiol by *Rhodococcus* species. J. Ferment. Bioeng..

[84] Stafford D.E., Yanagimachi K.S., Lessard P.A., Rijhwani S.K., Sinskey A.J., Stephanopoulos G. (2002). Optimizing bioconversion pathways through systems analysis and metabolic engineering. Proc. Natl. Acad. Sci. USA.

[85] Amanullah A., Hewitt C.J., Nienow A.W., Lee C., Chartrain M., Buckland B.C., Drew S.W., Woodley J.M. (2002). Fed-batch bioconversion of indene to *cis*-indandiol. Enzym. Microb. Technol..

[86] Priefert H., O’Brien X.M., Lessard P.A., Dexter A.F., Choi E.E., Tomic S., Nagpal G., Cho J.J., Agosto M., Yang L. (2004). Indene bioconversion by a toluene inducible dioxygenase of *Rhodococcus* sp. I24. Appl. Microbiol. Biotechnol..

[87] Solyanikova I.P., Emelyanova E.V., Borzova O.V., Golovleva L.A. (2016). Benzoate degradation by *Rhodococcus opacus* 1CP after dormancy: Characterization of dioxygenases involved in the process. J. Environ. Sci. Health Part B.

[88] Yang X., Xie F., Zhang G., Shi Y., Qian S. (2008). Purification, characterization, and substrate specificity of two 2,3-dihydroxybiphenyl 1,2-dioxygenase from *Rhodococcus* sp. R04, showing their distinct stability at various temperature. Biochimie.

[89] Xiong F., Shuai J.J., Peng R.H., Tian Y.S., Zhao W., Yao Q.H., Xiong A.S. (2011). Expression, purification and functional characterization of a recombinant 2,3-dihydroxybiphenyl-1,2-dioxygenase from *Rhodococcus rhodochrous*. Mol. Biol. Rep..

[90] Cha C.J. (2006). Metabolizing Alkyl-Substituted Catechols N75 Capable of metabolising alkyl-substituted catechols. J. Microbiol. Biotechnol..

[91] Matsumura E., Ooi S., Murakami S., Takenaka S., Aoki K. (2004). Constitutive synthesis, purification, and characterization of catechol 1,2-dioxygenase from the aniline-assimilating bacterium *Rhodococcus* sp. AN-22. J. Biosci. Bioeng..

[92] Deutz W.A., Fjaellman A.H.M., Ren S., Jourdat C., Witholt B. (2001). Biotransformation of d-limonene to (+) *trans* carveol by toluene-grown *Rhodococcus opacus* PWD4 cells. Appl. Environ. Microbiol..

[93] Thompson M.L., Marriott R., Dowle A., Grogan G. (2010). Biotransformation of β-myrcene to geraniol by a strain of *Rhodococcus erythropolis* isolated by selective enrichment from hop plants. Appl. Microbiol. Biotechnol..

[94] Van Der Geize R., Hessels G.I., Van Gerwen R., Van Der Meijden P., Dijkhuizen L. (2002). Molecular and functional characterization of kshA and kshB, encoding two components of 3-ketosteroid 9α-hydroxylase, a class IA monooxygenase, in *Rhodococcus erythropolis* strain SQ1. Mol. Microbiol..

[95] Li K., Wang J., Wu K., Zheng D., Zhou X., Han W., Wan N., Cui B., Chen Y. (2017). Enantioselective synthesis of 1,2,3,4-tetrahydroquinoline-4-ols and 2,3-dihydroquinolin-4(1:H)-ones via a sequential asymmetric hydroxylation/diastereoselective oxidation process using *Rhodococcus equi* ZMU-LK19. Org. Biomol. Chem..

[96] Sheldon R.A., Brady D. (2019). Broadening the Scope of Biocatalysis in Sustainable Organic Synthesis. ChemSusChem.

[97] Stompor M., Kałużny M., Żarowska B. (2016). Biotechnological methods for chalcone reduction using whole cells of *Lactobacillus*, *Rhodococcus* and *Rhodotorula* strains as a way to produce new derivatives. Appl. Microbiol. Biotechnol..

[98] Chen B.-S., Medici R., van der Helm M.P., van Zwet Y., Gjonaj L., van der Geest R., Otten L.G., Hanefeld U. (2018). *Rhodococcus* strains as source for ene-reductase activity. Appl. Microbiol. Biotechnol..

[99] Riedel A., Mehnert M., Paul C.E., Westphal A.H., van Berkel W.J.H., Tischler D. (2015). Functional characterization and stability improvement of a “thermophilic-like” ene-reductase from *Rhodococcus opacus* 1CP. Front. Microbiol..

[100] Hummel W., Schütte H., Schmidt E., Wandrey C., Kula M.R. (1987). Isolation of l-phenylalanine dehydrogenase from *Rhodococcus* sp. M4 and its application for the production of l-phenylalanine. Appl. Microbiol. Biotechnol..

[101] Bradshaw C.W., Wong C.H., Hummel W., Kula M.R. (1991). Enzyme-catalyzed asymmetric synthesis of (*S*)-2-amino-4-phenylbutanoic acid and (*R*)-2-hydroxy-4-phenylbutanoic acid. Bioorg. Chem..

[102] Sharma M., Mangas-Sanchez J., Turner N.J., Grogan G. (2017). NAD(P)H-Dependent Dehydrogenases for the Asymmetric Reductive Amination of Ketones: Structure, Mechanism, Evolution and Application. Adv. Synth. Catal..

[103] Ye L.J., Toh H.H., Yang Y., Adams J.P., Snajdrova R., Li Z. (2015). Engineering of amine dehydrogenase for asymmetric reductive amination of ketone by evolving *Rhodococcus* phenylalanine dehydrogenase. ACS Catal..

[104] Liu J., Pang B.Q.W., Adams J.P., Snajdrova R., Li Z. (2017). Coupled Immobilized Amine Dehydrogenase and Glucose Dehydrogenase for Asymmetric Synthesis of Amines by Reductive Amination with Cofactor Recycling. ChemCatChem.

[105] Knaus T., Böhmer W., Mutti F.G. (2017). Amine dehydrogenases: Efficient biocatalysts for the reductive amination of carbonyl compounds. Green Chem..

[106] Takeuchi K., Koike K., Ito S. (1990). Production of *cis*-unsaturated hydrocarbons by a strain of *Rhodococcus* in repeated batch culture with a phase-inversion, hollow-fiber system. J. Biotechnol..

[107] Araki H., Hagihara H., Takigawa H., Tsujino Y., Ozaki K. (2016). Novel Genes Encoding Hexadecanoic Acid Δ6-Desaturase Activity in a *Rhodococcus* sp.. Curr. Microbiol..

[108] Araki H., Hagiwara H., Kotani S., Yukiharu T., Takikawa H. (2004). Rhodococcus Fatty Acid Δ6-Desaturase Genes Useful for Production of *cis*-6-Hexadecenoic Acid.

[109] Bhalla T.C., Kumar V., Kumar V. (2018). Enzymes of aldoxime-nitrile pathway for organic synthesis. Rev. Environ. Sci. Biotechnol..

[110] Bhalla T.C., Kumar V., Kumar V., Thakur N. (2018). Savitri Nitrile Metabolizing Enzymes in Biocatalysis and Biotransformation. Appl. Biochem. Biotechnol..

[111] Kato Y., Ryoko O., Asano Y. (2000). Distribution of aldoxime dehydratase in microorganisms. Appl. Environ. Microbiol..

[112] Betke T., Higuchi J., Rommelmann P., Oike K., Nomura T., Kato Y., Asano Y., Gröger H. (2018). Biocatalytic Synthesis of Nitriles through Dehydration of Aldoximes: The Substrate Scope of Aldoxime Dehydratases. ChemBioChem.

[113] Yamaguchi T., Yamamoto K., Asano Y. (2014). Identification and characterization of CYP79D16 and CYP71AN24 catalyzing the first and second steps in l-phenylalanine-derived cyanogenic glycoside biosynthesis in the Japanese apricot, *Prunus mume* Sieb. et Zucc. Plant Mol. Biol..

[114] Gong J.S., Shi J.S., Lu Z.M., Li H., Zhou Z.M., Xu Z.H. (2017). Nitrile-converting enzymes as a tool to improve biocatalysis in organic synthesis: Recent insights and promises. Crit. Rev. Biotechnol..

[115] Asano Y., Kato Y. (1998). *Z*-phenylacetaldoxime degradation by a novel aldoxime dehydratase from *Bacillus* sp. strain OxB-1. FEMS Microbiol. Lett..

[116] Kato Y., Tsuda T., Asano Y. (1999). Nitrile hydratase involved in aldoxime metabolism from *Rhodococcus* sp. strain YH3-3. J. Mol. Catal. B Enzym..

[117] Metzner R., Okazaki S., Asano Y., Gröger H. (2014). Cyanide-free enantioselective synthesis of nitriles: Synthetic proof of a biocatalytic concept and mechanistic insights. ChemCatChem.

[118] Kato Y., Yoshida S., Xie S.-X., Asano Y. (2004). Aldoxime Dehydratase Co-existing with Nitrile Hydratase and Amidase in the Iron-Type Nitrile Hydratase-Producer *Rhodococcus* sp. N-771. J. Biosci. Bioeng..

[119] Xie S.X., Kato Y., Komeda H., Yoshida S., Asano Y. (2003). A Gene Cluster Responsible for Alkylaldoxime Metabolism Coexisting with Nitrile Hydratase and Amidase in *Rhodococcus globerulus* A-4. Biochemistry.

[120] Betke T., Rommelmann P., Oike K., Asano Y., Gröger H. (2017). Cyanide-Free and Broadly Applicable Enantioselective Synthetic Platform for Chiral Nitriles through a Biocatalytic Approach. Angew. Chem. Int. Ed..

[121] Kato Y., Nakamura K., Sakiyama H., Mayhew S.G., Asano Y. (2000). Novel heme-containing lyase, phenylacetaldoxime dehydratase from *Bacillus* sp. strain OxB-1: Purification, characterization, and molecular cloning of the gene. Biochemistry.

[122] BASF S.E. (2015). Method for Biocatalytic Production of Nitriles from Oximes and Oxime Dehydratases Usable Therein.

[123] Asano Y., Faber K., Fessner W.-D., Turner N.J. (2015). Nitrile and amide hydrolysis and enzymatic synthesis of amides and peptides. Biocatalysis in Organic Synthesis 1.

[124] Prasad S., Bhalla T.C. (2010). Nitrile hydratases (NHases): At the interface of academia and industry. Biotechnol. Adv..

[125] Nagasawa T., Shimizu H., Yamada H. (1993). The superiority of the third-generation catalyst, *Rhodococcus rhodochrous* J1 nitrile hydratase, for industrial production of acrylamide. Appl. Microb. Biotechnol..

[126] Nagasawa T., Takeuchi K., Nardi-Dei V., Mihara Y., Yamada H. (1991). Optimum culture conditions for the production of cobalt-containing nitrile hydratase by *Rhodococcus rhodochrous* J1. Appl. Microbiol. Biotechnol..

[127] Li B., Su J., Tao J. (2011). Enzyme and process development for production of nicotinamide. Org. Process Res. Dev..

[128] Nagasawa T., Mathew C.D., Mauger J., Yamada H. (1988). Nitrile Hydratase-Catalyzed Production of Nicotinamide from 3-Cyanopyridine in *Rhodococcus rhodochrous* J1. Appl. Environ. Microbiol..

[129] Mauger J., Nagasawa T., Yamada H. (1988). Nitrile hydratase-catalyzed production of isonicotinamide, picolinamide and pyrazinamide from 4-cyanopyridine, 2-cyanopyridine and cyanopyrazine in *Rhodococcus rhodochrous* J1. J. Biotechnol..

[130] Meth-Cohn O., Wang M.X. (1997). An in-depth study of the biotransformation of nitriles into amides and/or acids using *Rhodococcus rhodochrous* AJ270. J. Chem. Soc. Perkin Trans. 1.

[131] Kashiwagi M., Fuhshuku K.I., Sugai T. (2004). Control of the nitrile-hydrolyzing enzyme activity in *Rhodococcus rhodochrous* IFO 15564: Preferential action of nitrile hydratase and amidase depending on the reaction condition factors and its application to the one-pot preparation of amides from aldehyd. J. Mol. Catal. B Enzym..

[132] Holtze M.S., Sørensen J., Hansen H.C.B., Aamand J. (2006). Transformation of the herbicide 2,6-dichlorobenzonitrile to the persistent metabolite 2,6-dichlorobenzamide (BAM) by soil bacteria known to harbour nitrile hydratase or nitrilase. Biodegradation.

[133] Tang R., Shen Y., Wang M., Zhai Y., Gao Q. (2017). Highly chemoselective and efficient production of 2,6-difluorobenzamide using *Rhodococcus ruber* CGMCC3090 resting cells. J. Biosci. Bioeng..

[134] Wang M.X., Lin S.J., Liu C.S., Zheng Q.Y., Li J.S. (2003). Nitrile biotransformations for highly efficient and enantioselective syntheses of electrophilic oxiranecarboxamides. J. Org. Chem..

[135] Pawar S.V., Yadav G.D. (2014). Enantioselective enzymatic hydrolysis of *rac*-mandelonitrile to (*R*)-mandelamide by nitrile hydratase immobilized on poly(vinyl alcohol)/chitosan-glutaraldehyde support. Ind. Eng. Chem. Res..

[136] Wang Y.J., Xue Y.P., Liu Z.Q., Zheng Y.G., Zheng R.C. (2012). Screening, cultivation, and biocatalytic performance of *Rhodococcus boritolerans* FW815 with strong 2,2-dimethylcyclopropanecarbonitrile hydratase activity. J. Ind. Microbiol. Biotechnol..

[137] Crosby J., Moilliet J., Parratt J.S., Turner N.J. (1994). Regioselective hydrolysis of aromatic dinitriles using a whole cell catalyst. J. Chem. Soc. Perkin Trans. 1.

[138] Matoishi K., Sano A., Imai N., Yamazaki T., Yokoyama M., Sugai T., Ohta H. (1998). *Rhodococcus rhodochrous* IFO 15564-mediated hydrolysis of alicyclic nitriles and amides: Stereoselectivity and use for kinetic resolution and asymmetrization. Tetrahedron Asymmetry.

[139] Shen Y., Wang M., Li X., Zhang J., Sun H., Luo J. (2012). Highly efficient synthesis of 5-cyanovaleramide by *Rhodococcus ruber* CGMCC3090 resting cells. J. Chem. Technol. Biotechnol..

[140] Yasukawa K., Hasemi R., Asano Y. (2011). Dynamic kinetic resolution of α-aminonitriles to form chiral α-amino acids. Adv. Synth. Catal..

[141] Kubáč D., Kaplan O., Elišáková V., Pátek M., Vejvoda V., Slámová K., Tóthová A., Lemaire M., Gallienne E., Lutz-Wahl S. (2008). Biotransformation of nitriles to amides using soluble and immobilized nitrile hydratase from *Rhodococcus erythropolis* A4. J. Mol. Catal. B Enzym..

[142] Vervisch K., D’Hooghe M., Rutjes F.P.J.T., De Kimpe N. (2012). Chemical and enzymatic synthesis of 2-(2-carbamoylethyl)- and 2-(2-carboxyethyl)aziridines and their conversion into δ-lactams and γ-lactones. Org. Lett..

[143] Ohta H. (1996). Stereochemistry of enzymatic hydrolysis of nitriles. Chimia (Aarau).

[144] Sugai T., Yamazaki T., Yokoyama M., Ohta H. (1997). Biocatalysis in Organic Synthesis: The use of nitrile- and amide-hydrolyzing microorganisms. Biosci. Biotech. Biochem..

[145] Kakeya H., Sakai N., Sugai T., Ohta H. (1991). Microbial hydrolysis as a potent method for the preparation of optically active nitriles, amides and carboxylic acids. Tetrahedron Lett..

[146] Beard T., Cohen M.A., Parratt J.S., Turner N.J., Crosby J., Moilliet J. (1993). Stereoselective hydrolysis of nitriles and amides under mild conditions using a whole cell catalyst. Tetrahedron Asymmetry.

[147] Layh N., Stolz A., Böhme J., Effenberger F., Knackmuss H.J. (1994). Enantioselective hydrolysis of racemic naproxen nitrile and naproxen amide to *S*-naproxen by new bacterial isolates. J. Biotechnol..

[148] Wang M.X., Wu Y. (2003). Nitrile biotransformations for the synthesis of enantiomerically enriched Baylis-Hillman adducts. Org. Biomol. Chem..

[149] Ao Y.F., Wang D.X., Zhao L., Wang M.X. (2015). Synthesis of quaternary-carbon-containing and functionalized enantiopure pentanecarboxylic acids from biocatalytic desymmetrization of *meso*-cyclopentane-1,3-dicarboxamides. Chem. Asian J..

[150] Yokoyama M., Kashiwagi M., Iwasaki M., Fuhshuku K.I., Ohta H., Sugai T. (2004). Realization of the synthesis of α,α-disubstituted carbamylacetates and cyanoacetates by either enzymatic or chemical functional group transformation, depending upon the substrate specificity of *Rhodococcus* amidase. Tetrahedron Asymmetry.

[151] Osprian I., Fechter M.H., Griengl H. (2003). Biocatalytic hydrolysis of cyanohydrins: An efficient approach to enantiopure α-hydroxy carboxylic acids. J. Mol. Catal. B Enzym..

[152] Wegman M.A., Heinemann U., Van Rantwijk F., Stolz A., Sheldon R.A. (2001). Hydrolysis of d,l-phenylglycine nitrile by new bacterial cultures. J. Mol. Catal. B Enzym..

[153] Lin Z.J., Zheng R.C., Zheng Y.G., Shen Y.C. (2011). Biosynthesis of 2-amino-2,3-dimethylbutyramide by nitrile hydratase from a newly isolated cyanide-resistant strain of *Rhodococcus qingshengii*. Biotechnol. Lett..

[154] Wang M.X., Lin S.J. (2002). Practical and convenient enzymatic synthesis of enantiopure α-amino acids and amides. J. Org. Chem..

[155] Morán-Ramallal R., Liz R., Gotor V. (2007). Bacterial preparation of enantiopure unactivated aziridine-2-carboxamides and their transformation into enantiopure nonnatural amino acids and vic-diamines. Org. Lett..

[156] Leng D.H., Wang D.X., Huang Z.T., Wang M.X. (2010). Highly efficient and enantioselective biotransformations of β-lactam carbonitriles and carboxamides and their synthetic applications. Org. Biomol. Chem..

[157] Leng D.H., Wang D.X., Pan J., Huang Z.T., Wang M.X. (2009). Highly efficient and enantioselective biotransformations of racemic azetidine-2-carbonitriles and their synthetic applications. J. Org. Chem..

[158] Kakeya H., Sakai N., Sano A., Yokoyama M., Sugai T., Ohta H. (1991). Microbiol hydrolysis of 3-substituted glutaronitriles. Chem. Lett..

[159] Crosby J.A., Parrattt J.S., Turner N.J. (1992). Enzymatic hydrolysis of prochiral dinitriles. Tetrahedron Asymmetry.

[160] Maddrell S.J., Turner N.J., Kerridge A., Willetts A.J., Crosby J. (1996). Nitrile Hydratase Enzymes in Organic Synthesis. Tetrahedron Lett..

[161] Kinfe H.H., Chhiba V., Frederick J., Bode M.L., Mathiba K., Steenkamp P.A., Brady D. (2009). Enantioselective hydrolysis of β-hydroxy nitriles using the whole cell biocatalyst *Rhodococcus rhodochrous* ATCC BAA-870. J. Mol. Catal. B Enzym..

[162] Wu Z.L., Li Z.Y. (2003). Biocatalytic asymmetric hydrolysis of (±)-β-hydroxy nitriles by *Rhodococcus* sp. CGMCC 0497. J. Mol. Catal. B Enzym..

[163] Yokoyama M., Imai N., Sugai T., Ohta H. (1996). Preparation of both enantiomers of methyl 3-benzoyloxypentanoate by enzyme-catalysed hydrolysis of corresponding racemic nitrile and amide. J. Mol. Catal. B Enzym..

[164] Vink M.K.S., Schortinghuis C.A., Luten J., Van Maarseveen J.H., Schoemaker H.E., Hiemstra H., Rutjes F.P.J.T. (2002). A stereodivergent approach to substituted 4-hydroxypiperidines. J. Org. Chem..

[165] Méndez-Sánchez D., López-Iglesias M., Gotor-Fernández V. (2016). Hydrolases in Organic Chemistry. Recent Achievements in the Synthesis of Pharmaceuticals. Curr. Org. Chem..

[166] Ma Y., Yu H., Pan W., Liu C., Zhang S., Shen Z. (2010). Identification of nitrile hydratase-producing *Rhodococcus ruber* TH and characterization of an amiE-negative mutant. Bioresour. Technol..

[167] Hall M., Faber K., Tasnadi G., Faber K., Fessner W.-D., Turner N.J. (2015). Hydrolysis of Amides. Biocatalysis in Organic Synthesis 1.

[168] Ismailsab M., Monisha T.R., Reddy P.V., Santoshkumar M., Nayak A.S., Karegoudar T.B. (2017). Biotransformation of aromatic and heterocyclic amides by amidase of whole cells of *Rhodococcus* sp. MTB5: Biocatalytic characterization and substrate specificity. Biocatal. Biotransform..

[169] Wang M.X., Lu G., Ji G.J., Huang Z.T., Meth-Cohn O., Colby J. (2000). Enantioselective biotransformations of racemic α-substituted phenylacetonitriles and phenylacetamides using *Rhodococcus* sp. AJ270. Tetrahedron Asymmetry.

[170] Effenberger F., Graef B.W., Oßwald S. (1997). Preparation of (*S*)-naproxen by enantioselective hydrolysis of racemic naproxen amide with resting cells of *Rhodococcus erythropolis* MP50 in organic solvents. Tetrahedron Asymmetry.

[171] Snell D., Colby J. (1999). Enantioselective hydrolysis of racemic ibuprofen amide to *S*-(+)-ibuprofen by *Rhodococcus* AJ270. Enzym. Microb. Technol..

[172] Wu Z.L., Li Z.Y. (2003). Practical synthesis of optically active α,α-disubstituted malonamic acids through asymmetric hydrolysis of malonamide derivatives with *Rhodococcus* sp. CGMCC 0497. J. Org. Chem..

[173] Zhang L.B., Wang D.X., Wang M.X. (2011). Microbial whole cell-catalyzed desymmetrization of prochiral malonamides: Practical synthesis of enantioenriched functionalized carbamoylacetates and their application in the preparation of unusual α-amino acids. Tetrahedron.

[174] Zhang L.B., Wang D.X., Zhao L., Wang M.X. (2012). Synthesis and application of enantioenriched functionalized α-tetrasubstituted α-amino acids from biocatalytic desymmetrization of prochiral α-aminomalonamides. J. Org. Chem..

[175] Chen P., Gao M., Wang D.X., Zhao L., Wang M.X. (2012). Enantioselective biotransformations of racemic and meso pyrrolidine-2,5-dicarboxamides and their application in organic synthesis. J. Org. Chem..

[176] Chen P., Gao M., Wang D.X., Zhao L., Wang M.X. (2012). Practical biocatalytic desymmetrization of *meso*-*N*-heterocyclic dicarboxamides and their application in the construction of aza-sugar containing nucleoside analogs. Chem. Commun..

[177] Ao Y.F., Zhang L.-B., Wang Q.Q., Wang D.X., Wang M.X. (2018). Biocatalytic Desymmetrization of Prochiral 3-Aryl and 3-Arylmethyl Glutaramides: Different Remote Substituent Effect on Catalytic Efficiency and Enantioselectivity. Adv. Synth. Catal..

[178] Venhoff N., Setzer B., Melkaoui K., Walker U.A. (2007). Mitochondrial toxicity of tenofovir, emtricitabine and abacavir alone and in combination with additional nucleoside reverse transcriptase inhibitors. Antivir. Ther..

[179] Taylor S.J.C., Sutherland A.G., Lee C., Wisdom R., Thomas S., Roberts S.M., Evans C. (1990). Chemoenzymatic Synthesis of (-)-carbovir utilising a whole cell catalysed resolution of 2-azabicyclo[2.2.1]hept-5-en-3-one. J. Chem. Soc. Chem. Commun..

[180] Assaf Z., Eger E., Vitnik Z., Fabian W.M.F., Ribitsch D., Guebitz G.M., Faber K., Hall M. (2014). Identification and application of enantiocomplementary lactamases for Vince lactam derivatives. ChemCatChem.

[181] Fukuta Y., Koizumi S., Komeda H., Asano Y. (2010). A new aryl acylamidase from *Rhodococcus* sp. strain Oct1 acting on ω-lactams: Its characterization and gene expression in *Escherichia coli*. Enzym. Microb. Technol..

[182] Asano Y., Fukuta Y., Yoshida Y., Komeda H. (2008). The screening, characterization, and use of ω-laurolactam hydrolase: A new enzymatic synthesis of 12-aminolauric acid. Biosci. Biotechnol. Biochem..

[183] Nagasawa T., Nakamura T., Yamada H. (1990). Production of acrylic acid and methacrylic acid using *Rhodococcus rhodochrous* J1 nitrilase. Appl. Microbiol. Biotechnol..

[184] Luo H., Wang T., Yu H., Yang H., Shen Z. (2006). Expression and catalyzing process of the nirilase in *Rhodococcus rhodochrous* tg1-A6. Mod. Chem. Ind..

[185] Webster N.A., Ramsden D.K., Hughes J. (2001). Comparative characterisation of two *Rhodococcus* species as potential biocatalysts for ammonium acrylate production. Biotechnol. Lett..

[186] Bhalla T.C., Miura A., Wakamoto A., Ohba Y., Furuhashi K. (1992). Asymmetric hydrolysis of α-aminonitriles to optically active amino acids by a nitrilase of *Rhodococcus rhodochrous* PA-34. Appl. Microbiol. Biotechnol..

[187] Mathew C.D., Nagasawa T., Kobayashi M., Yamada H. (1988). Nitrilase-Catalyzed Production of Nicotinic Acid from 3-Cyanopyridine in *Rhodococcus rhodochrous* J1. Appl. Environ. Microbiol..

[188] Prasad S., Misra A., Jangir V.P., Awasthi A., Raj J., Bhalla T.C. (2007). A propionitrile-induced nitrilase of *Rhodococcus* sp. NDB 1165 and its application in nicotinic acid synthesis. World J. Microbiol. Biotechnol..

[189] Martínková L., Vesela A.B., Faber K., Fessner W.-D., Turner N.J. (2015). Hydrolysis of nitriles to carboxylic acids. Biocatalysis in Organic Synthesis 1.

[190] Gong J.S., Lu Z.M., Li H., Shi J.S., Zhou Z.M., Xu Z.H. (2012). Nitrilases in nitrile biocatalysis: Recent progress and forthcoming research. Microb. Cell Fact..

[191] Nigam V.K., Arfi T., Kumar V., Shukla P. (2017). Bioengineering of Nitrilases towards Its Use as Green Catalyst: Applications and Perspectives. Indian J. Microbiol..

[192] Archelas A., Iacazio G., Kotik M., Patel R.M. (2016). Epoxide hydrolase and its application in organic synthesis. Green Biocatalysis.

[193] Osprian I., Kroutil W., Mischitz M., Faber K. (1997). Biocatalytic resolution of 2-methyl-2-(aryl)alkyloxiranes using novel bacterial epoxide hydrolases. Tetrahedron Asymmetry.

[194] Wandel U., Mischitz M., Kroutil W., Faber K. (1995). Highly selective asymmetric hydrolysis of 2,2-disubstituted epoxides using lyophilized cells of *Rhodococcus* sp. NCIMB 11216. J. Chem. Soc. Perkin Trans. 1.

[195] Osprian I., Stampfer W., Faber K. (2000). Selectivity enhancement of epoxide hydrolase catalyzed resolution of 2,2-disubstituted oxiranes by substrate modification. J. Chem. Soc. Perkin Trans. 1.

[196] Woo J.H., Kang K.M., Kwon T.H., Park N.H., Lee E.Y. (2015). Isolation, identification and characterization of marine bacteria exhibiting complementary enantioselective epoxide hydrolase activity for preparing chiral chlorinated styrene oxide derivatives. J. Ind. Eng. Chem..

[197] Arand M., Hallberg B.M., Zou J., Bergfors T., Oesch F., Van der Werf M.J., De Bont J.A.M., Jones T.A., Mowbray S.L. (2003). Structure of *Rhodococcus erythropolis* limonene-1,2-epoxide hydrolase reveals a novel active site. EMBO J..

[198] Van Der Werf M.J., Orru R.V.A., Overkamp K.M., Swarts H.J., Osprian I., Steinreiber A., De Bont J.A.M., Faber K. (1999). Substrate specificity and stereospecificity of limonene-1,2-epoxide hydrolase from *Rhodococcus erythropolis* DCL14; an enzyme showing sequential and enantioconvergent substrate conversion. Appl. Microbiol. Biotechnol..

[199] Van der Werf M., Overkamp K., de Bont J. (1998). DCL14 Belongs to a Novel Class of Epoxide Hydrolases. J. Bacteriol..

[200] Zheng H., Reetz M.T. (2010). Manipulating the stereoselectivity of limonene epoxide hydrolase by directed evolution based in iterative saturation mutagenesis. J. Am. Chem. Soc..

[201] Bottalla A.L., Ibrahim-Ouali M., Santelli M., Furstoss R., Archelas A. (2007). Epoxide hydrolase-catalysed kinetic resolution of a spiroepoxide, a key building block of various 11-heterosteroids. Adv. Synth. Catal..

[202] Kroutil W., Mischitz M., Faber K. (1997). Derazemisation of (±)-2,3-disubstituted oxiranes via biocatalytic hydrolysis Using Bacterial Epoxide Hydrolases: Kinetics of an Enantioconvergent Process. J. Chem. Soc. Perkin Trans. 1.

[203] Steinreiber A., Mayer S.F., Saf R., Faber K. (2001). Biocatalytic asymmetric and enantioconvergent hydrolysis of trisubstituted oxiranes. Tetrahedron Asymmetry.

[204] Steinreiber A., Mayer S.F., Faber K. (2001). Asymmetric total synthesis of a beer-aroma constituent based on enantioconvergent biocatalytic hydrolysis of trisubstituted epoxides. Synthesis (Stuttg).

[205] Edegger K., Mayer S.F., Steinreiber A., Faber K. (2004). Chemo-enzymatic enantio-convergent asymmetric synthesis of (*R*)-(+)-Marmin. Tetrahedron.

[206] Liu Z., Li Y., Xu Y., Ping L., Zheng Y. (2007). Cloning, sequencing, and expression of a novel epoxide hydrolase gene from *Rhodococcus opacus* in *Escherichia coli* and characterization of enzyme. Appl. Microbiol. Biotechnol..

[207] Glueck S.M., Fabian W.M.F., Faber K., Mayer S.F. (2004). Biocatalytic asymmetric rearrangement of a methylene-interrupted *bis*-epoxide: Simultaneous control of four asymmetric centers through a biomimetic reaction cascade. Chem. A Eur. J..

[208] Ueberbacher B.T., Oberdorfer G., Gruber K., Faber K. (2009). Epoxide-hydrolase-initiated hydrolysis/rearrangement cascade of a methylene-interrupted *bis*-epoxide yields chiral THF moieties without involvement of a “Cyclase”. ChemBioChem.

[209] Hellström H., Steinreiber A., Mayer S.F., Faber K. (2001). Bacterial epoxide hydrolase-catalyzed resolution of a 2,2-disubstituted oxirane: Optimization and upscaling. Biotechnol. Lett..

[210] Simeó Y., Faber K. (2006). Selectivity enhancement of enantio- and stereo-complementary epoxide hydrolases and chemo-enzymatic deracemization of (±)-2-methylglycidyl benzyl ether. Tetrahedron Asymmetry.

[211] Fuchs M., Simeo Y., Ueberbacher B.T., Mautner B., Netscher T., Faber K. (2009). Enantiocomplementary chemoenzymatic asymmetric synthesis of (*R*)- And (*S*)-chromanemethanol. Eur. J. Org. Chem..

[212] Mischitz M., Faber K. (1996). Chemo-enzymatic synthesis of linalool oxide. Synlett.

[213] Steinreiber A., Edegger K., Mayer S.F., Faber K. (2001). (2*R*,5*S*)-Pityol through enzyme-triggered ring closure. Tetrahedron Asymmetry.

[214] Zhang Y., Pan J., Luan Z.J., Xu G.C., Park S., Xu J.H. (2014). Cloning and characterization of a novel esterase from *Rhodococcus* sp. for highly enantioselective synthesis of a chiral cilastatin precursor. Appl. Environ. Microbiol..

[215] Luan Z.J., Yu H.L., Ma B.-D., Qi Y.K., Chen Q., Xu J.H. (2016). Dramatically improved performance of an esterase for cilastatin synthesis by cap domain engineering. Ind. Eng. Chem. Res..

[216] Pogorevc M., Strauss U.T., Hayn M., Faber K. (2000). Novel carboxyl esterase preparations for the resolution of linalyl acetate. Mon. Chem..

[217] Akutsu-Shigeno Y., Adachi Y., Yamada C., Toyoshima K., Nomura N., Uchiyama H., Nakajima-Kambe T. (2006). Isolation of a bacterium that degrades urethane compounds and characterization of its urethane hydrolase. Appl. Microbiol. Biotechnol..

[218] Lorenzen J., Janke R., Waldow A., Qoura F., Loll B., Brück T. (2017). *Rhodococcus erythropolis* Oleate Hydratase: A New Member in the Oleate Hydratase Family Tree—Biochemical and Structural Studies. ChemCatChem.

[219] Engleder M., Pavkov-Keller T., Emmerstorfer A., Hromic A., Schrempf S., Steinkellner G., Wriessnegger T., Leitner E., Strohmeier G.A., Kaluzna I. (2015). Structure-Based Mechanism of Oleate Hydratase from *Elizabethkingia meningoseptica*. ChemBioChem.

[220] Park A.K., Lee G.H., Kim D.W., Jang E.H., Kwon H.T., Chi Y.M. (2018). Crystal structure of oleate hydratase from *Stenotrophomonas* sp. KCTC 12332 reveals conformational plasticity surrounding the FAD binding site. Biochem. Biophys. Res. Commun..

[221] Volkov A., Khoshnevis S., Neumann P., Herrfurth C., Wohlwend D., Ficner R., Feussner I. (2013). Crystal structure analysis of a fatty acid double-bond hydratase from *Lactobacillus acidophilus*. Acta Crystallogr. Sect. D Biol. Crystallogr..

[222] Busch H., Tonin F., Alvarenga N., van den Broek M., Lu S., Daran J.-M., Hanefeld U., Hagedoorn P.-L. Exploring the abundance of oleate hydratases in the genus *Rhodococcus*—Discovery of novel enzymes with complementary substrate scope.

[223] Brueck T., Lorenzen J. (2018). A Process for the Cell-Free Enzymtaic Production of 10-Hydroxystearic Acid (10-HSA) from Bio-Based Oils for Lubricant Formulation.

[224] Holland H.L., Gu J.X. (1998). Preparation of (*R*)-3-hydroxy-3-alkylbutanolides by biocatalytic hydration of 3-alkyl-2-butenolides using *Rhodococcus rhodochrous*. Biotechnol. Lett..

[225] Chen B.-S., Resch V., Otten L.G., Hanefeld U. (2015). Enantioselective Michael Addition of Water. Chem. A Eur. J..

[226] Busch H., Alvarenga N., Abdelraheem E., Hoek M., Hagedoorn P.-L., Hanefeld U. (2019). Re-investigation of hydration potential of *Rhodococcus* whole-cell biocatalysts towards Michael acceptors. ChemCatChem.

[227] He Y.C., Tao Z.C., Zhang D.P., Yang Z.X., Gao S., Ma C.L. (2015). Biotransformation of 1,3-propanediol cyclic sulfate and its derivatives to diols by *Rhodococcus* sp.. Biotechnol. Lett..

[228] Gadler P., Faber K. (2007). New enzymes for biotransformations: Microbial alkyl sulfatases displaying stereo- and enantioselectivity. Trends Biotechnol..

[229] Pogorevc M., Kroutil W., Wallner S.R., Faber K. (2002). Enantioselective stereoinversion in the kinetic resolution of *rac*-*sec*-alkyl sulfate esters by hydrolysis with an alkylsulfatase from *Rhodococcus ruber* DSM 44541 furnishes homochiral products. Angew. Chem. Int. Ed..

[230] Pogorevc M., Faber K. (2003). Purification and characterization of an inverting stereo- and enantioselective *sec*-alkylsulfatase from the gram-positive bacterium *Rhodococcus ruber* DSM 44541. Appl. Environ. Microbiol..

[231] Pogorevc M., Faber K. (2002). Enantioselective stereoinversion of *sec*-alkyl sulfates by an alkylsulfatase from *Rhodococcus ruber* DSM 44541. Tetrahedron Asymmetry.

[232] Pogorevc M., Strauss U.T., Riermeier T., Faber K. (2002). Selectivity-enhancement in enantioselective hydrolysis of *sec*-alkyl sulfates by an alkylsulfatase from *Rhodococcus ruber* DSM 44541. Tetrahedron Asymmetry.

[233] He Y.C., Ma C.L., Yang Z.X., Zhou M., Xing Z., Ma J.T., Yu H.L. (2013). Highly enantioselective oxidation of phenyl methyl sulfide and its derivatives into optically pure (*S*)-sulfoxides with *Rhodococcus* sp. CCZU10-1 in an n-octane-water biphasic system. Appl. Microbiol. Biotechnol..

[234] Li A.T., Zhang J.D., Xu J.H., Lu W.Y., Lin G.Q. (2009). Isolation of *Rhodococcus* sp. strain ECU0066, a new sulfide monooxygenase-producing strain for asymmetric sulfoxidation. Appl. Environ. Microbiol..

[235] Li A.T., Zhang J.D., Yu H.L., Pan J., Xu J.H. (2011). Significantly improved asymmetric oxidation of sulfide with resting cells of *Rhodococcus* sp. in a biphasic system. Process Biochem..

[236] Lei B., Shiao-Chun T.U. (1996). Gene overexpression, purification, and identification of a desulfurization enzyme from *Rhodococcus* sp. strain IGTS8 as a sulfide/sulfoxide monooxygenase. J. Bacteriol..

